# Multicenter evaluation of prognostic nutritional index and systemic immune-inflammation index in predicting mortality among critically ill cardiovascular and cerebrovascular patients with varied glucose metabolism: a machine learning-based cohort study

**DOI:** 10.3389/fnut.2026.1703589

**Published:** 2026-02-03

**Authors:** Zhimin Li, Mingchen Xie, Haitao Wu, Tingxuan Wang, Shujie Huang, Jianhua Cheng

**Affiliations:** 1 Department of Neurosurgery, The Affiliated Hospital of Qingdao University, Qingdao, Shandong, China; 2 Department of Critical Care Medicine, Jishuitan Hospital, Beijing, China; 3 Department of Vascular Surgery, Qingdao Hospital, University of Health and Rehabilitation Sciences (Qingdao Municipal Hospital), Qingdao, Shandong, China

**Keywords:** critically ill patients, glucose metabolic status, machine learning, prognostic nutritional index, systemic immune-inflammation index

## Abstract

**Background:**

Critically ill patients with cardiovascular and cerebrovascular diseases face high mortality risks, necessitating precise prognostic tools. Current models lack granularity in assessing glucose metabolic subgroups, while isolated use of the Prognostic Nutritional Index (PNI) and Systemic Immune-Inflammation Index (SII) has limitations. This study evaluates their combined predictive value for mortality across glucose metabolic profiles using machine learning.

**Methods:**

We conducted a retrospective cohort study of 1,698 patients from the MIMIC-IV database (2008–2019), stratified by glucose metabolic status: normal glucose regulation (NGR), prediabetes (Pre-DM), and diabetes mellitus (DM). Prognostic associations and discrimination performance were evaluated using Cox regression, Kaplan–Meier analysis, and ROC curves. Machine learning models—including logistic regression, decision tree, random forest, XGBoost, and LightGBM—were developed based on Boruta-selected features to predict 28-day and 90-day all-cause mortality. Model performance was assessed using AUC, accuracy, and F1-score. To externally validate the machine learning models, we incorporated an independent cohort of critically ill cardiovascular and cerebrovascular patients (n = 1,194) from two tertiary hospitals in China: The Affiliated Hospital of Qingdao University and Qingdao Municipal Hospital.

**Results:**

Higher PNI was associated with reduced mortality, whereas elevated SII predicted higher mortality risk. The combined PNI-SII model outperformed individual indices across glucose subgroups, showing the best performance in Pre-DM patients (AUC = 0.775 for 28-day mortality). PNI’s protective effect was attenuated in the DM group, while SII remained consistently predictive. Machine learning models confirmed PNI and SII as top-ranking mortality predictors, particularly in NGR and Pre-DM populations. External validation demonstrated robust generalizability of the models, with comparable AUCs and calibration metrics across the independent Chinese cohort, supporting cross-center applicability.

**Conclusion:**

Integration of PNI and SII improves risk stratification and mortality prediction among critically ill patients with cardiovascular and cerebrovascular diseases, especially those with prediabetes. The machine learning models exhibited strong generalizability when externally validated using real-world data from two tertiary hospitals, underscoring their potential for broader clinical application and personalized decision-making.

## 1 Introduction

Cardiovascular and cerebrovascular diseases, as the leading causes of death and disability worldwide, have posed an extremely severe threat to public health (1, 2). In clinical practice, the special population of critically ill patients with cardiovascular and cerebrovascular diseases has attracted particular attention, characterized by complex and variable conditions, frequent complications, and a high all-cause mortality rate (3). Accurate prediction of all-cause mortality in such patients is of crucial significance for optimizing clinical treatment decisions, rationally allocating medical resources, and improving patient prognosis (4). However, due to the involvement of numerous complex pathophysiological mechanisms in cardiovascular and cerebrovascular diseases, which are influenced by the interaction of multiple factors, achieving accurate prediction of all-cause mortality in critically ill patients has long been one of the major challenges in the field of clinical research (5, 6). Glucose metabolic status plays a fundamental regulatory role in human physiological functions, and its dysregulation is closely linked to the onset, progression, and prognosis of cardiovascular and cerebrovascular diseases (7, 8). Clinically observed glucose metabolic states, including normoglycemia, impaired glucose tolerance, and diabetes mellitus, can induce damage to the cardiovascular and cerebrovascular system through multiple pathways—such as altering vascular endothelial function, affecting hemorheological properties, triggering oxidative stress responses, and regulating the release of inflammatory mediators (9–12). A large body of research has confirmed that compared with patients with normal glucose metabolism, those with cardiovascular and cerebrovascular diseases and abnormal glucose metabolism often exhibit poorer clinical outcomes, with significantly increased all-cause mortality (13, 14). However, refined predictive models for all-cause mortality in critically ill cardiovascular and cerebrovascular patients stratified by glucose metabolic status remain relatively scarce, failing to meet the urgent needs of clinical precision medicine. The systemic immune-inflammation index (SII), as a critical marker reflecting the body’s immune-inflammatory status, has increasingly been applied in clinical practice to assess disease progression and prognosis across various conditions. By incorporating quantitative changes in key blood cell components, SII multidimensionally captures the interplay between immunity and inflammation, thereby illustrating how the body’s immune-inflammatory state influences disease onset and progression. This provides clinicians with a quantifiable reference for evaluating disease severity (15, 16). Meanwhile, the prognostic nutritional index (PNI), which integrates serum albumin levels with lymphocyte counts, comprehensively reflects a patient’s nutritional status and underlying inflammatory activity. It has also been validated to be closely associated with the prognosis of cardiovascular and cerebrovascular diseases (17, 18). Despite their demonstrated predictive value in individual studies, the standalone use of SII or PNI for predicting all-cause mortality in critically ill cardiovascular and cerebrovascular patients has inherent limitations. Neither can fully or accurately encompass the multifaceted factors affecting patient outcomes, making them susceptible to predictive biases.

As information technology becomes ever more deeply integrated into the medical field, machine learning—with its exceptional capabilities in data mining, pattern recognition, and predictive analytics—has increasingly established itself as a powerful tool in medical research (19). Against this backdrop, the present study aims to leverage machine learning approaches for integrated assessment of PNI and SII, to develop predictive models for all-cause mortality in critically ill patients with cardiovascular and cerebrovascular diseases across distinct glucose metabolic states. This endeavor seeks to provide a more scientific, accurate, and clinically actionable decision-making basis for clinical practice, thereby enhancing medical management and improving prognostic outcomes in this patient cohort.

## 2 Methods and subjects

### 2.1 Data sources

This observational cohort study utilized a retrospective analysis of clinical data extracted from the Medical Information Mart for Intensive Care IV (MIMIC-IV v3.1) database. As a publicly accessible repository, MIMIC-IV encompasses comprehensive clinical records of over 190,000 patients and 450,000 hospital admissions documented at Beth Israel Deaconess Medical Center (BIDMC) in Boston, Massachusetts, USA, with a 12-year time span from 2008 to 2019. Notably, to safeguard patient privacy, MIMIC-IV has specifically de-identified admission timestamps (including exact admission dates, hospitalization sequences, and related information), rendering it impossible for researchers to determine the specific year of admission for individual patients. The study protocol was approved by the Institutional Review Board of the Massachusetts Institute of Technology (Certification ID: 66829613). Given the de-identified nature of the pre-existing medical records, the informed consent process was waived.

### 2.2 Population

**Figure 1:**
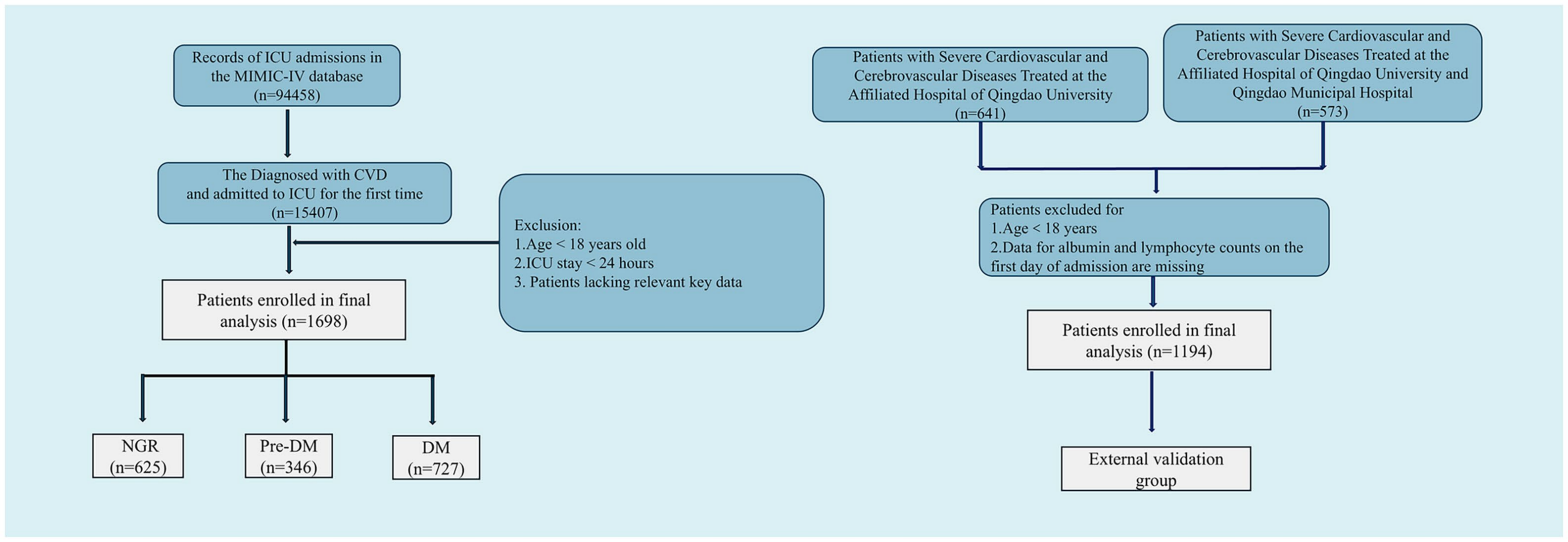
Flowchart of the selection of patients.

Critically ill patients with cardiovascular and cerebrovascular diseases were identified through manual review of ICD-9 and ICD-10 codes. In the MIMIC-IV database, ICD-9 and ICD-10 codes are discharge diagnostic codes assigned by clinicians upon completion of hospitalization. These codes have been standardized for billing, administrative, and epidemiological purposes, reflecting the final diagnosis for a specific hospitalization episode. Detailed specifications of the diagnostic codes are provided in Supplementary Table S1. Exclusion criteria were as follows: (1) age < 18 years at the time of initial admission; (2) ICU length of stay < 24 h; and (3) insufficient data, particularly regarding platelets, albumin, neutrophils, and lymphocytes. For critically ill subjects with multiple admissions, only the initial hospitalization episode was included in the analysis. A flowchart depicting the patient enrollment process is shown in Figure 1.

### 2.3 Data extraction

Data extraction was performed using Structured Query Language (SQL) via Navicat Premium (v16.3.11), with a focus on five domains:

Demographic data: age (years), gender (n, %).
Vital signs: heart rate (bpm, HR), respiratory rate (bpm, RR), mean arterial pressure (mmHg, MAP), oxygen saturation (%, SpO₂).
Laboratory parameters: hemoglobin (g/dL, Hb), platelets (10^9^/L, Plt), red blood cells (×10^6^/μL, RBC), white blood cells (K/μL, WBC), potassium (mEq/L), sodium (mEq/L), glucose (mg/dL), prothrombin time (s, PT), creatinine (mg/dL), blood urea nitrogen (mg/dL, BUN).
Comorbidities: hypertension (HTN), chronic obstructive pulmonary disease (COPD), liver cirrhosis (LC), acute kidney injury (AKI), chronic kidney disease (CKD), normal glucose regulation (NGR), prediabetes (Pre-DM), diabetes mellitus (DM).
Clinical severity scores: Sequential Organ Failure Assessment (SOFA), Acute Physiology and Chronic Health Evaluation II (APACHE II score), Charlson Comorbidity Index (CCI).

$$\begin{array}{l} \mathrm{PNI}:10\times \text{serum albumin}\;(g/\mathrm{dL})+0.005\times \text{total number of}\;\\ \text{peripheral blood lymphocytes}\;(/{\mathrm{mm}}^{3}). \end{array}$$

$$\begin{array}{l} \mathrm{SII}:\text{Platelet count}\;(\times 10^{9}/L)\times \text{Neutrophils}\;(\times 10^{9}/L)/\\ \text{lymphocytes}\;(\times 10^{9}/L). \end{array}$$

### 2.4 Subgroup classification criteria

Participants were categorized into three distinct cohorts based on established diagnostic criteria for glucose metabolism: normal glucose regulation (NGR), prediabetes (Pre-DM), and diabetes mellitus (DM). The NGR group consisted of individuals with an HbA1c level <5.7% and no prior history of diabetes mellitus. The Pre-DM cohort included patients with an HbA1c level ranging from 5.7% (inclusive) to 6.5% and no previous history of diabetes. The DM group comprised patients with a history of diabetes mellitus or an HbA1c level ≥6.5%.

Vital signs (heart rate, respiratory rate, body temperature) and all other variables were defined as the first measured values obtained within 24 h of ICU admission. This approach ensured the capture of baseline physiological status prior to any interventions in the intensive care unit (ICU) that might alter these parameters. Variables with a missing data proportion of ≥20% were excluded to reduce potential bias; for variables with a missing data proportion of <20%, multiple imputation was used to fill in the missing values.

### 2.5 Outcome measures

The primary outcome was 28-day all-cause mortality, and the secondary outcome was 90-day all-cause mortality.

### 2.6 Statistical analysis

Participants were categorized into high and low groups based on tertiles of PNI and SII (PNI: <36.10, 36.10–43.40, >43.40; SII: <1013.4, 1013.40–2257.70, >2257.70). The top tertile was defined as “high,” while the lower two tertiles were classified as “low.” The normality of continuous variables was evaluated via the Shapiro–Wilk test. Normally distributed variables, reported as mean ± standard deviation (SD), were analyzed using the independent-samples t-test or one-way analysis of variance (ANOVA). Non-normally distributed variables, reported as median [interquartile range (IQR)], were compared using the Wilcoxon rank-sum test. Categorical variables, reported as counts (percentages), were analyzed using the χ^2^ test or Fisher’s exact test. Multicollinearity was evaluated using the variance inflation factor (VIF), with variables exhibiting a VIF > 5 excluded from multivariable analyses. Kaplan–Meier (KM) curves were constructed to estimate cumulative all-cause mortality risk. Cox proportional hazards regression models were implemented in three successive steps: Model 1 (unadjusted); Model 2 (adjusted for age and sex); and Model 3 (adjusted for age, sex, HTN, AKI, LC, HB, RBC, WBC, PT, BUN). Covariates were selected based on clinically relevant indicators and those that demonstrated significance in univariable analyses, with collinearity controlled (VIF < 5). Restricted cubic spline (RCS) analysis was performed to explore the dose–response relationships of PNI and SII with outcomes. The proportional hazards assumption was verified via Schoenfeld residuals. Receiver operating characteristic (ROC) curves were utilized to compare the predictive performance of various indicators, as measured by area under the curve (AUC), sensitivity, and specificity. Lastly, sensitivity analyses were conducted to assess the robustness of the findings, excluding patients with incomplete covariate data and those who experienced at least one episode of hypoglycemia. For the machine learning-driven predictive modeling, the Boruta algorithm was employed to rank feature importance for 28-day mortality. The dataset was then randomly partitioned into a training set (80%) and a testing set (20%).

Based on the selected features, five models were constructed: logistic regression (LR), random forest (RF), extreme gradient boosting (XGBoost), light gradient boosting machine (LightGBM), and categorical boosting (CatBoost). These algorithms were deliberately chosen to represent complementary modeling strategies across different levels of complexity and interpretability. Logistic regression, as a classical linear model, provides transparency and ease of clinical interpretation, facilitating understanding of the direction and magnitude of associations. In contrast, tree-based ensemble and gradient boosting methods (RF, XGBoost, LightGBM, and CatBoost) are well suited for modeling nonlinear relationships and higher-order feature interactions, while maintaining robustness to multicollinearity, outliers, and moderate sample sizes. Collectively, this combination allows for a balanced evaluation of predictive performance, model stability, and clinical interpretability, while mitigating overfitting risk. Model performance was evaluated via metrics such as the area under the receiver operating characteristic curve (AUC), accuracy, specificity, sensitivity, and F1 score. The optimal model, as determined by these metrics, was further elucidated using SHapley Additive exPlanations (SHAP) to identify key predictive factors. All analyses were conducted using Python 3.9.12, SPSS 26.0, and DecisionLnnc 1.0. Statistical significance was defined as a two-tailed *p*-value < 0.05.

## 3 Results

### 3.1 Baseline characteristics of the patients

This study enrolled 1,698 critically ill patients with cardiovascular and cerebrovascular cardiovascular and cerebrovascular diseases, comprising 1,469 cases in the survival group and 229 cases in the non-survival group. Baseline characteristics are summarized in Table 1. Significant intergroup differences were observed in PNI and SII (both *p* < 0.001). The median PNI in the overall population was 39.8 (interquartile range [IQR], 33.9–45.5), with a higher value in the survival group (40.4, 34.5–45.8) and a significantly lower value in the non-survival group (36.1, 30.7–42.1), suggesting that a reduced PNI is associated with an elevated risk of death. The overall median SII was 1492.7 (IQR, 791.3–2861.1), which was substantially higher in the non-survival group (2195.3, 1123.4–4036.0) than in the survival group (1403.5, 772.8–2674.7), indicating that excessive activation of immune inflammation exacerbates poor prognosis. Regarding demographic characteristics, the overall median age was 69.0 years (IQR, 59.0–78.0), with the non-survival group being significantly older (71.0 years, 62.0–82.0, *p* = 0.007). Females accounted for 38.2% (649/1698) of the total population, with no significant intergroup difference (*p* = 0.215). For comorbidities, significant differences were noted in the distribution of HTN, LC, and AKI (all *p* < 0.05). The proportion of AKI in the non-survival group was 67.7% (155/229), markedly higher than that in the survival group (44.3%, 651/1469). The non-survival group also had a significantly higher proportion of LC (10.0%, 23/229) compared with the survival group (4.6%, 68/1469), implying that hepatic decompensation may disrupt metabolic and immune homeostasis, thereby increasing the risk of death. In terms of vital signs and laboratory parameters, HR, RR, and MAP differed significantly between the two groups (all *p* < 0.05). The non-survival group exhibited a faster HR (91 bpm, 77–102), a higher RR (20 bpm, 18–26), and a lower MAP (84 mmHg, 71–98), reflecting an imbalance in circulatory and respiratory compensation. Additionally, significant differences were observed in Hb, RBC, and WBC (all p < 0.05), with the non-survival group showing lower levels of Hb and RBC, and higher levels of WBC, blood glucose, creatinine, and blood urea nitrogen. Among clinical scoring systems, the SOFA score, Acute Physiology and APACHE II score, and CCI were significantly higher in the non-survival group (all *p* < 0.001).

**Table 1:** Baseline characteristics according to 28-day mortality.

Variable	Overall (*N* = 1,698)	Survivors (*N* = 1,469)	Non-survivors (*N* = 229)	*p*
PNI	39.8 (33.9–45.5)	40.4 (34.5–45.8)	36.1 (30.7–42.1)	<0.001
SII	1492.7 (791.3–2861.1)	1403.5 (772.8–2674.7)	2195.3 (1123.4–4036.0)	<0.001
Demographics				
Age (years)	69.0 (59.0–78.0)	68.0 (59.0–78.0)	71.0 (62.0–82.0)	0.007
Female, *n* (%)	649 (38.2%)	553 (37.6%)	96 (41.9%)	0.215
Vital signs				
HR (bpm)	85.0 (73.0–100.0)	84.0 (72.0–99.0)	91.0 (77.0–102.0)	<0.001
RR(bpm)	19.0 (16.0–24.0)	19.0 (16.0–23.0)	20.0 (18.0–26.0)	<0.001
MAP (mmHg)	87.0 (76.0–100.0)	88.0 (77.0–100.0)	84.0 (71.0–98.0)	0.004
SpO_2_ (%)	97.0 (95.0–100.0)	97.0 (95.0–100.0)	97.0 (93.0–100.0)	0.188
Comorbidities				
HTN, *n* (%)	649 (38.2%)	576 (39.2%)	73 (31.9%)	0.034
COPD, *n* (%)	247 (14.5%)	204 (13.9%)	43 (18.8%)	0.051
LC, *n* (%)	91 (5.4%)	68 (4.6%)	23 (10.0%)	<0.001
AKI, *n* (%)	806 (47.5%)	651 (44.3%)	155 (67.7%)	<0.001
CKD, *n* (%)	443 (26.1%)	374 (25.5%)	69 (30.1%)	0.134
Glucose metabolism state				0.024
NGR, n (%)	625 (36.8%)	558 (38.0%)	67 (29.3%)	
Pr-DM, n (%)	346 (20.4%)	299 (20.4%)	47 (20.5%)	
DM, n (%)	727 (42.8%)	612 (41.7%)	115 (50.2%)	
Laboratory measurements				
Hb(g/dL)	11.6 (9.8–13.3)	11.6 (9.9–13.3)	11.1 (9.1–13.1)	0.008
Plt(10⁹/L)	205.0 (152.0–262.0)	207.0 (155.0–261.0)	198.0 (133.0–273.0)	0.132
RBC(m/Ul)	3.9 (3.4–4.5)	4.0 (3.4–4.5)	3.7 (3.1–4.4)	0.003
WBC(K/Ul)	11.2 (8.2–15.4)	10.9 (8.1–14.8)	13.7 (9.7–18.9)	<0.001
Potassium(mEq/L)	4.1 (3.8–4.6)	4.1 (3.8–4.6)	4.2 (3.9–4.8)	0.052
Sodium(mEq/L)	139.0 (136.0–141.0)	139.0 (136.0–141.0)	138.0 (135.0–142.0)	0.593
Glucose(mg/dL)	134.0 (107.0–188.0)	131.0 (105.0–183.0)	153.0 (123.0–219.0)	<0.001
PT (s)	13.2 (12.0–15.4)	13.1 (12.0–15.3)	14.6 (12.5–18.1)	<0.001
Creatinine(mg/dL)	1.1 (0.8–1.6)	1.0 (0.8–1.6)	1.3 (0.9–2.1)	<0.001
Urea Nitrogen(mg/dL)	21.0 (14.0–35.0)	20.0 (14.0–32.0)	27.0 (17.0–48.0)	<0.001
Clinical scores				
SOFA	4.0 (2.0–7.0)	4.0 (2.0–7.0)	6.0 (4.0–9.0)	<0.001
APACHE II score	17.0 (12.0–23.0)	16.0 (12.0–22.0)	22.0 (17.0–27.0)	<0.001
CCI	6.0 (4.0–8.0)	6.0 (4.0–8.0)	7.0 (5.0–9.0)	<0.001

PNI, prognostic nutritional index; HR, heart rate; RR, respiratory rate; MAP, mean arterial pressure; HTN, hypertension; COPD, chronic obstructive pulmonary disease; LC, liver cirrhosis; AKI: acute kidney injury; CKD, chronic kidney disease; NGR, normal glucose regulation; Pre-DM, pre-diabetes mellitus; DM, diabetes mellitus; HB, hemoglobin; PLT, platelet; RBC, red blood cell; WBC, white blood cell.

### 3.2 Association between PNI and SII and mortality in patients with different glucose metabolic status

#### 3.2.1 KM curves to examine the association of PNI and SII alone and in combination with 28-day mortality in patients with different glucose metabolic status

**Figure 2:**
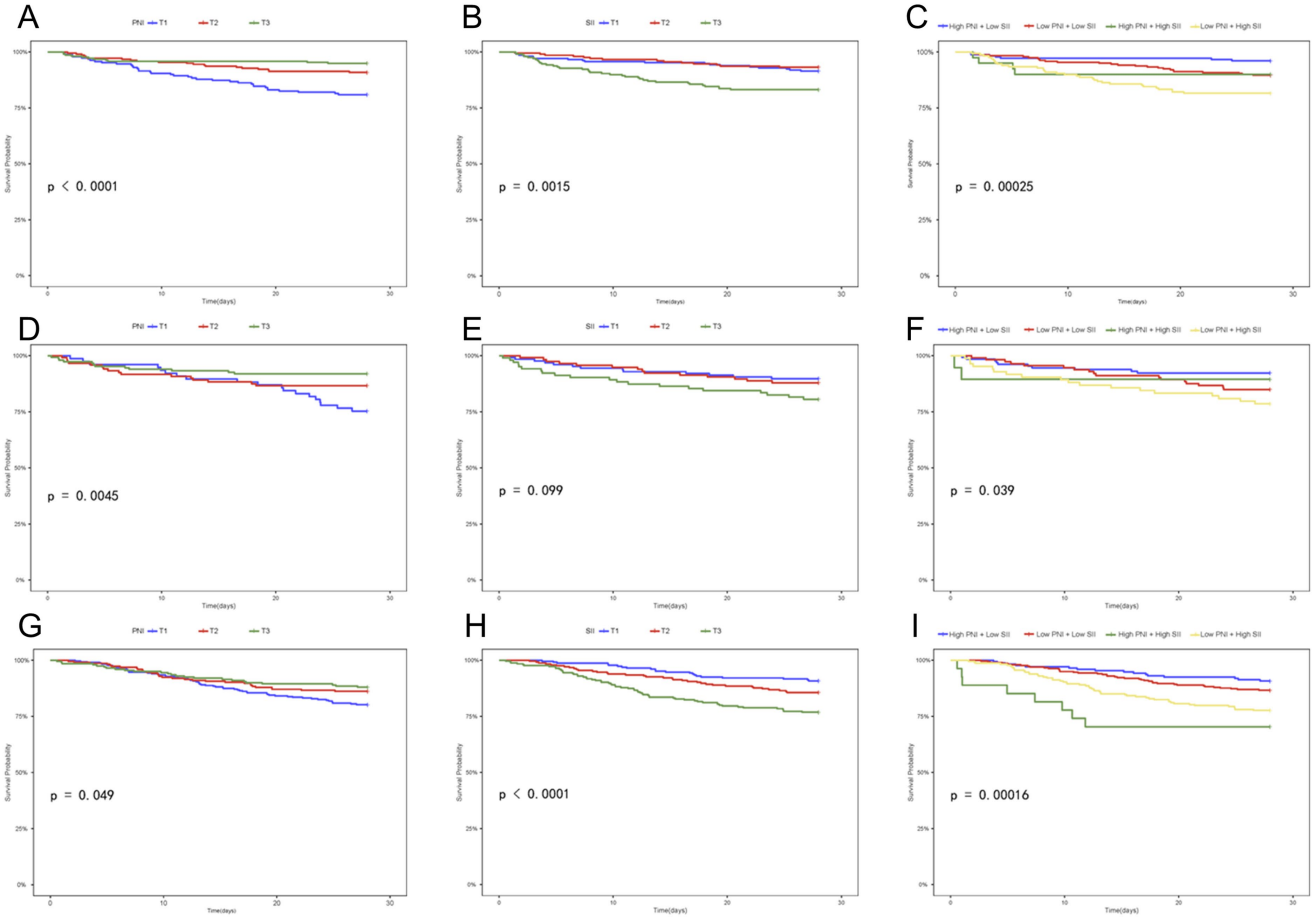
Kaplan–Meier curves of PNI, SII, and their combination for 28-day mortality. **(A–C)** Patients with NGR; **(D–F)** patients with Pre-DM; **(G–I)** patients with DM.

The Kaplan–Meier survival curves presented herein demonstrate the impact of indicator-based stratification on all-cause mortality risk in critically ill patients with cardiovascular and cerebrovascular diseases from multiple dimensions: Figure 2A illustrates the survival curves for the NGR subgroup in PNI-stratified analysis (*p* < 0.0001), where the T3 group exhibited the highest survival probability and the T1 group the lowest, indicating a strong association between higher PNI and lower mortality risk in NGR patients. Figure 2B shows the NGR subgroup in SII-stratified analysis (*p* = 0.0015), with the T3 group having the lowest survival probability, suggesting that a high inflammatory state predicts poorer prognosis in NGR patients. Figure 2C displays the NGR subgroup in combined PNI + SII phenotype stratification (*p* = 0.0025), where the “high PNI + low SII” phenotype showed the best survival and the “low PNI + high SII” phenotype the worst, validating the synergistic protective effect of “high nutrition - low inflammation” and highlighting the superior predictive value of combined assessment over a single indicator.

Figure 2D indicates that in the Pre-DM subgroup of PNI-stratified analysis (*p* = 0.0045), the T3 group still maintained a survival advantage, though the degree of curve separation was weaker than in the NGR subgroup, suggesting that during Pre-DM, the protective effect of PNI persists but is modified by metabolic disturbances, with slightly reduced efficacy. In Figure 2E, the Pre-DM subgroup in SII-stratified analysis (*p* = 0.99) showed minimal curve separation, reflecting that the stability of SII’s predictive power declines when Pre-DM is in a transitional metabolic state, possibly due to more complex interactions between inflammation and glucose metabolic disorders. Figure 2F shows that in the Pre-DM subgroup of combined PNI + SII phenotype stratification (*p* = 0.039), phenotypic stratification remained effective but with a weaker degree of separation than in the NGR subgroup, indicating that the predictive power of the combined phenotype persists in Pre-DM but is modified by metabolic abnormalities. In Figure 2G, the DM subgroup in PNI-stratified analysis (*p* = 0.049) showed a narrower degree of curve separation; however, the T3 group still outperformed the T1 group, indicating that the predictive value of PNI is not completely lost in DM patients. Figure 2H presents the DM subgroup in SII-stratified analysis (*p* < 0.0001), where the T3 group showed a sharp decline in survival, suggesting that the lethal effect of high SII is amplified in DM patients, with the chronic inflammatory background of diabetes enhancing the prognostic value of SII. Figure 2F illustrates the DM subgroup in combined PNI + SII phenotype stratification (*p* = 0.0016), where the “low PNI + high SII” phenotype carried the highest mortality risk and the “high PNI + low SII” phenotype maintained a high survival probability, indicating that even in DM patients, combined phenotypic stratification can refine prognostic assessment, with more pronounced effects due to greater variations in PNI and SII.

Taken together, these Kaplan–Meier curves validate, from the perspective of survival analysis, the following: 1. PNI and SII have predictive value for the prognosis of critically ill patients with cardiovascular and cerebrovascular diseases; 2. this value is modulated by glucose metabolic status; 3. the predictive performance of combined assessment is superior.

#### 3.2.2 COX curves to explore the associations of PNI and SII with 28-day and 90-day mortality in patients with different glucose metabolism status

This study investigated the associations of the PNI combined with the SII with 28-day and 90-day all-cause mortality in critically ill patients with cardiovascular and cerebrovascular diseases using Cox regression models, with stratified analyses performed according to glucose metabolic status. The results are presented in Supplementary Table S2: In the analysis of 28-day all-cause mortality, PNI was significantly negatively correlated with the risk of death (i.e., a higher PNI was associated with a lower risk). In the total population, the risks in the T2 and T3 subgroups were both lower than that in the T1 reference subgroup, with stable associations observed in Model 1 and Model 3. A stronger protective effect was noted in NGR patients (Model 1: T2, HR = 0.457; T3, HR = 0.251), with T3 remaining significant in Model 3. In Pre-DM patients, only T3 was associated with a significant reduction in risk (Model 1: HR = 0.318; Model 3: HR = 0.472), while T2 approached significance. In DM patients, the protective effect of T3 was present in Model 1 (HR = 0.591) but was attenuated in Model 3 (HR = 0.957), with a significant trend observed across all populations. In contrast, SII was positively correlated with the risk of death (i.e., a higher SII was associated with a higher risk). In the total population, only T3 was significantly higher than T1 (Model 1: HR = 2.360; Model 3: HR = 2.072). In NGR patients, T3 was associated with an elevated risk (Model 1: HR = 2.089; Model 3: HR = 1.905). In Pre-DM patients, T3 was significant in Model 2 (HR = 2.045). In DM patients, the risk in T3 was particularly prominent (Model 1: HR = 2.770; Model 3: HR = 2.126). No significant association was observed for T2 in all populations, but a significant trend was noted. The 90-day analysis revealed a similar trend: regarding the negative correlation with PNI, in the total population, T3 was associated with a lower risk (Model 1: HR = 0.666; Model 3: HR = 0.590), and T2 also reached significance in Model 3 (HR = 0.755). In NGR patients, T3 exerted a strong protective effect (Model 1: HR = 0.482; Model 3: HR = 0.415), with T2 being significant in Model 3 (HR = 0.606). In Pre-DM patients, only T3 was associated with a significant reduction in risk (Model 1: HR = 0.473; Model 3: HR = 0.423), with a significant trend. No significant association was found in DM patients. For the positive correlation with SII, T3 was associated with a higher risk than T1 in the total population, NGR patients, and DM patients (total population: Model 1, HR = 1.842; Model 3, HR = 1.925; NGR patients: Model 1, HR = 2.049; Model 3, HR = 1.850; DM patients: Model 1, HR = 1.726; Model 3, HR = 1.830), with significant trends observed. In Pre-DM patients, T3 was significant in Model 3 (HR = 2.415), accompanied by a significant trend. All analyses indicated that these associations were modulated by glucose metabolic status, with the predictive value of PNI being diminished in DM patients.

In summary, PNI was negatively associated with 28-day and 90-day all-cause mortality (i.e., a higher PNI corresponded to a lower risk of death), with stable associations in the total population, NGR patients, and Pre-DM patients. In DM patients, the association was significant for 28-day mortality but not for 90-day mortality. SII was positively associated with the risk of death (i.e., a higher SII was linked to a higher risk of death), with stable associations across all populations, particularly in NGR patients, DM patients, and the total population. Most associations remained significant after adjustment for covariates, suggesting that PNI and SII could serve as potential predictors of mortality risk in critically ill patients with cardiovascular and cerebrovascular diseases across different glucose metabolic statuses.

#### 3.2.3 COX curve to explore the association between PNI combined with SII and 28-day and 90-day mortality in patients with different glucose metabolism status

This study investigated the associations of combined PNI and SII with 28-day and 90-day all-cause mortality in critically ill patients with cardiovascular and cerebrovascular diseases using Cox regression models, with stratification by glucose metabolic status. The grouping criteria were as follows: Group 1 (low SII and high PNI), Group 2 (low SII and low PNI), Group 3 (high SII and high PNI), and Group 4 (high SII and low PNI). Results are presented in Table 2.

**Table 2:** The association of the combination of PNI and SII with all-cause mortality.

Variables	Model 1		Model 2		Model 3	
	HR (95%CI)	*P*	HR (95%CI)	*P*	HR (95%CI)	*P*
28-day mortality						
Overall						
Group 1	1.00 (Reference)		1.00 (Reference)		1.00 (Reference)	
Group 2	1.861 (1.242 ~ 2.788)	0.003	1.812 (1.209 ~ 2.717)	0.004	1.596 (1.038 ~ 2.453)	0.033
Group 3	2.622 (1.403 ~ 4.899)	0.003	2.749 (1.479 ~ 5.141)	0.002	2.557 (1.361 ~ 4.805)	0.004
Group 4	3.267 (2.204 ~ 4.843)	<0.001	3.193 (2.153 ~ 4.735)	<0.001	2.573 (1.694 ~ 3.909)	<0.001
P for trend	1.421 (1.275 ~ 1.585)	<0.001	1.416 (1.270 ~ 1.580)	<0.001	1.333 (1.188 ~ 1.495)	<0.001
Patients with NGR						
Group 1	1.00 (Reference)		1.00 (Reference)		1.00 (Reference)	
Group 2	2.683 (1.160 ~ 6.202)	0.021	2.599 (1.122 ~ 6.022)	0.026	2.311 (1.004 ~ 5.533)	0.030
Group 3	2.678 (0.784 ~ 9.150)	0.116	2.808 (0.821 ~ 9.603)	0.100	2.951 (0.848 ~ 10.270)	0.089
Group 4	5.037 (2.218 ~ 11.440)	<0.001	4.864 (2.137 ~ 11.068)	<0.001	4.111 (1.764 ~ 9.579)	0.001
P for trend	1.545 (1.258 ~ 1.899)	<0.001	1.533 (1.247 ~ 1.844)	<0.001	1.479 (1.187 ~ 1.842)	<0.001
Patients with Pre-DM						
Group 1	1.00 (Reference)		1.00 (Reference)		1.00 (Reference)	
Group 2	1.980 (0.907 ~ 4.325)	0.086	2.087 (0.951 ~ 4.579)	0.066	1.739 (0.721 ~ 4.190)	0.218
Group 3	1.452 (0.318 ~ 6.625)	0.630	1.571 (0.342 ~ 7.222)	0.562	1.374 (0.284 ~ 6.655)	0.693
Group 4	2.965 (1.369 ~ 6.424)	0.006	3.003 (1.386 ~ 6.508)	0.005	2.538 (1.081 ~ 5.960)	0.032
P for trend	1.370 (1.087 ~ 1.727)	0.008	1.371 (1.089 ~ 1.727)	0.007	1.304 (1.014 ~ 1.677)	0.038
Patients with DM						
Group 1	1.00 (Reference)		1.00 (Reference)		1.00 (Reference)	
Group 2	1.475 (0.826 ~ 2.634)	0.189	1.430 (0.800 ~ 2.555)	0.227	1.276 (0.689 ~ 2.364)	0.439
Group 3	2.394 (1.109 ~ 4.025)	0.001	2.299 (1.833 ~ 4.081)	0.001	1.956 (1.496 ~ 3.493)	0.004
Group 4	2.619 (1.493 ~ 4.592)	0.001	2.538 (1.446 ~ 4.454)	0.001	2.000 (1.090 ~ 3.667)	0.025
P for trend	1.366 (1.169 ~ 1.595)	<0.001	1.358 (1.162 ~ 1.587)	<0.001	1.270 (1.074 ~ 1.501)	0.005
90-day mortality						
Overall						
Group 1	1.00 (Reference)		1.00 (Reference)		1.00 (Reference)	
Group 2	1.359 (0.943 ~ 1.960)	0.100	1.327 (0.920 ~ 1.913)	0.130	1.386 (0.942 ~ 2.038)	0.097
Group 3	2.521 (1.389 ~ 4.574)	0.002	2.636 (1.452 ~ 4.788)	0.001	2.689 (1.471 ~ 4.914)	0.001
Group 4	2.621 (1.405 ~ 4.908)	<0.001	2.954 (1.358 ~ 4.812)	<0.001	2.917 (1.312 ~ 5.030)	0.001
P for trend	1.249 (1.130 ~ 1.382)	<0.001	1.240 (1.121 ~ 1.371)	<0.001	1.222 (1.101 ~ 1.356)	<0.001
Patients with NGR						
Group 1	1.00 (Reference)		1.00 (Reference)		1.00 (Reference)	
Group 2	1.543 (0.761 ~ 3.128)	0.229	1.480 (0.729 ~ 3.005)	0.278	1.606 (0.780 ~ 3.308)	0.198
Group 3	2.385 (0.747 ~ 7.619)	0.142	2.559 (0.800 ~ 8.181)	0.113	2.697 (0.824 ~ 8.830)	0.101
Group 4	3.084 (1.530 ~ 6.219)	0.002	2.887 (1.428 ~ 5.836)	0.003	2.850 (1.391 ~ 5.842)	0.004
P for trend	1.436 (1.190 ~ 1.733)	<0.001	1.413 (1.170 ~ 1.706)	<0.001	1.382 (1.135 ~ 1.683)	0.001
Patients with Pre-DM						
Group 1	1.00 (Reference)		1.00 (Reference)		1.00 (Reference)	
Group 2	1.514 (0.727 ~ 3.153)	0.268	1.543 (0.740 ~ 3.217)	0.247	1.534 (0.674 ~ 3.488)	0.308
Group 3	1.319 (0.292 ~ 5.964)	0.719	1.353 (0.299 ~ 6.128)	0.695	1.510 (0.324 ~ 7.037)	0.600
Group 4	2.275 (1.104 ~ 4.686)	0.026	2.313 (1.121 ~ 4.769)	0.023	2.410 (1.084 ~ 5.357)	0.031
P for trend	1.282 (1.033 ~ 1.591)	0.024	1.287 (1.037 ~ 1.597)	0.022	1.306 (1.033 ~ 1.650)	0.026
Patients with DM						
Group 1	1.00 (Reference)		1.00 (Reference)		1.00 (Reference)	
Group 2	1.206 (0.708 ~ 2.056)	0.491	1.125 (0.659 ~ 1.920)	0.667	1.104 (0.624 ~ 1.950)	0.734
Group 3	2.276 (1.470 ~ 4.301)	0.004	2.262 (1.436 ~ 4.252)	0.003	2.777 (1.213 ~ 4.160)	0.016
Group 4	2.436 (1.690 ~ 4.733)	0.014	2.332 (1.623 ~ 4.633)	0.011	2.832 (1.513 ~ 4.360)	0.009
P for trend	1.138 (1.091 ~ 1.316)	0.003	1.142 (1.104 ~ 1.331)	0.003	1.100 (1.061 ~ 1.292)	0.002

Mode1 1: unadjusted. Model 2: adjusted for age and sex. Model 3: adjusted for age, sex, HTN, AKI, LC, HB, RBC, WBC, PT, Creatinine and BUN.

For 28-day mortality analysis: In Model 1, the mortality risk in Group 3 (HR = 2.659) and Group 4 (HR = 2.171) was significantly higher than that in the reference group (*p* < 0.05), with a dose–response effect observed (trend HR = 1.30, *p* < 0.001). After adjusting for age, sex, and other confounding factors (Model 3), Group 3 (HR = 1.50) and Group 4 (HR = 1.41) remained significant, though the trend strength weakened (HR = 1.15, *p* = 0.002). In subgroup analyses, Model 3 for NGR patients showed a significantly elevated risk only in Group 4 (HR = 1.81, *p* = 0.034); for Pre-DM patients, Group 3 exhibited a significant risk (HR = 2.04, *p* = 0.005); however, no statistically significant differences were observed among all groups in DM patients after adjustment. For 90-day mortality analysis: In Model 3 for the overall population, Group 3 (HR = 1.46) and Group 4 (HR = 1.40) maintained significant associations (*p* < 0.01), with the trend persisting (HR = 1.13, *p* = 0.001). In NGR patients, Model 3 revealed significantly increased risks in Group 3 (HR = 1.57) and Group 4 (HR = 1.75) (*p* < 0.05). For Pre-DM patients, Group 3 (HR = 1.57) and Group 4 (HR = 1.72) approached significance (*p* = 0.043–0.050). In DM patients, only Group 4 showed a significant risk (HR = 1.45, *p* = 0.047), with the trend failing to reach statistical significance (*p* = 0.083).

In conclusion, the comprehensive assessment combining PNI and SII was independently and positively associated with short-term (28-day) and medium-term (90-day) mortality in critically ill patients with cardiovascular and cerebrovascular diseases, particularly exhibiting more stable predictive performance in NGR and Pre-DM patients. Mortality risk increased with increasing group severity (Groups 3–4), indicating a significant dose–response effect. Although some associations weakened after adjusting for confounding factors, the trends remained robust in the overall population and non-diabetic subgroups. Notably, the associations were significantly attenuated in DM patients after multivariable adjustment (the 28-day trend disappeared, and only a single group remained significant at 90 days), suggesting that hyperglycemia may interfere with the predictive efficacy of inflammation-nutrition indices.

#### 3.2.4 RCS curve to explore the association of PNI and SII with 28-day and 90-day mortality in patients with different glucose metabolism status

**Figure 3:**
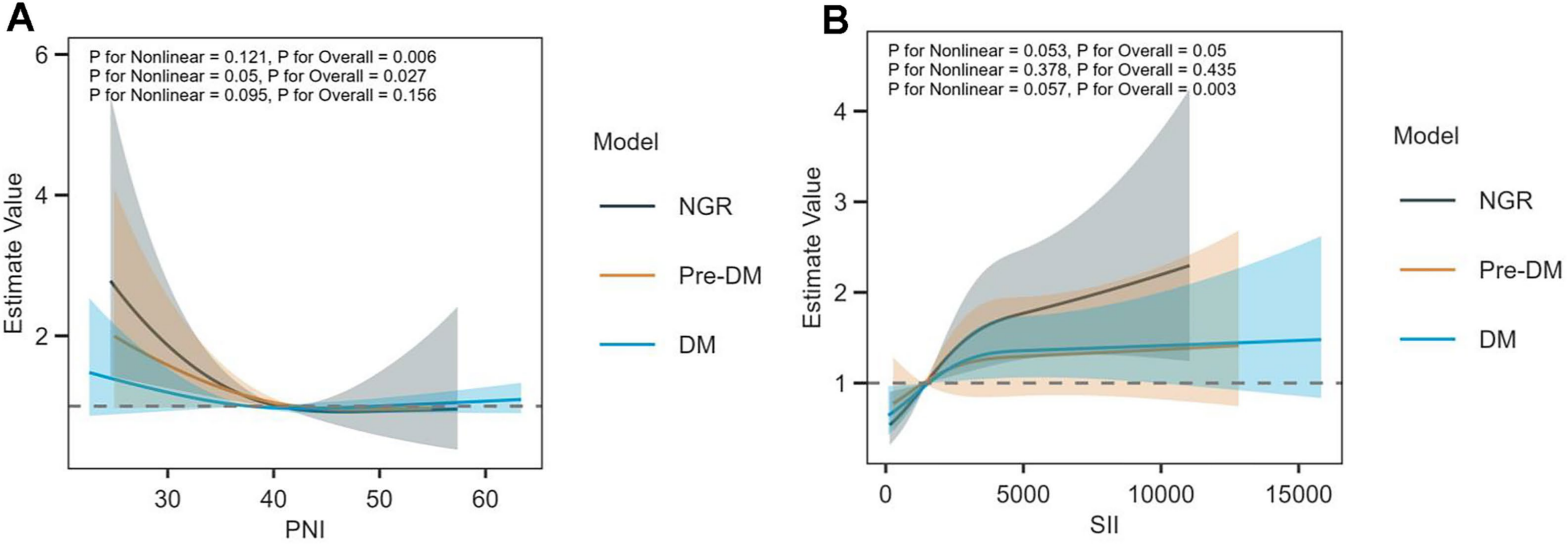
Multivariable-adjusted restricted cubic spline analyses of PNI and SII for 28-day mortality. Adjusted for covariates as in Table 2. **(A)** PNI and **(B)** SII.

For PNI (Figure 3A), the curve exhibited an “initial decline followed by plateauing” pattern, indicating a non-linear inverse association with all-cause mortality: at low PNI levels, an increase in PNI resulted in a rapid drop in risk estimates (significantly reduced mortality risk); at high PNI levels, the rate of decline in risk estimates slowed with further increases in PNI. Regarding subgroup variations, the reduction in risk estimates was more pronounced in the NGR subgroup, suggesting that PNI exerts a more prominent protective effect in critically ill patients with cardiovascular and cerebrovascular diseases and normal glucose metabolism. In contrast, the curves for the Pre-DM and DM subgroups were flatter, potentially due to glucose metabolic disturbances attenuating PNI’s nutritional-immune protective effects. For SII (Figure 3B), the curve displayed an “initial rise followed by continuous elevation” pattern, indicating a non-linear positive association with all-cause mortality: at low SII levels, an increase in SII induced a rapid rise in risk estimates (significantly increased mortality risk); at high SII levels, further elevation of SII led to a sustained rise in risk estimates (further increased mortality risk, with no obvious plateau phase). In terms of subgroup differences, in the high SII range, risk estimates in the DM subgroup were significantly higher than those in the NGR and Pre-DM subgroups, suggesting that the risk-exacerbating effect of elevated SII was more intense in diabetic patients. This phenomenon is potentially linked to glucose metabolic disturbances aggravating immune-inflammatory imbalance.

In summary, the RCS curve clearly showed that the protective effect of PNI on the risk of death and the promoting effect of SII on the risk of death both showed nonlinear changes with their own levels and glucose metabolism status.

#### 3.2.5 proportional hazards hypothesis test of cox proportional hazards regression model

Proportional hazards assumption tests for the combined PNI and SII subgroups were conducted in critically ill patients with cardiovascular and cerebrovascular diseases across different glucose metabolic statuses (Supplementary Tables S4–S6), with results as follows: In the NGR population: The subgroup (Group) test yielded a *p*-value of 0.3582 (>0.05), satisfying the proportional hazards assumption, indicating that the impact of subgrouping on mortality risk remained constant throughout the follow-up period. The global test (GLOBAL) showed a *p*-value of 0.0889 (>0.05), confirming the overall stability of the model, thus validating the reliability of the Cox model results. In the Pre-DM population: The Group test returned a *p*-value of 0.1738 (>0.05), indicating that mortality risk did not vary over time, with the hazard ratios for high-risk subgroups remaining stable from short-term to long-term follow-up. The GLOBAL test yielded a *p*-value of 0.2109 (>0.05), suggesting no time-dependent bias; the combined effect exhibited temporal stability, consistent with its background of high predictive value. In the DM population: The Group test showed a *p*-value of 0.0989 (>0.05, approaching the critical value), largely satisfying the assumption (the combined effect may exhibit slight variations in the late follow-up period due to chronic diabetic complications but remains generally acceptable). However, the GLOBAL test yielded a *p*-value of 0.0255 (<0.05), violating the assumption, potentially due to interference from other variables (e.g., AKI). Nevertheless, the stability of the PNI + SII subgrouping itself was not significantly impaired, and the model results retain reference value.

#### 3.2.6 Sensitivity analysis

Several sensitivity analyses were performed to evaluate the robustness of our findings. First, after excluding 46 patients who experienced at least one hypoglycemic episode during ICU hospitalization, the results of Cox proportional hazards regression analyses remained consistent with those of the original analysis (Supplementary Table S8). Second, following the exclusion of 423 participants with missing data, the association between the combined PNI and SII and prognosis in critically ill patients with cardiovascular and cerebrovascular diseases remained aligned with the primary outcome (Supplementary Table S7). These sensitivity analyses confirmed the reliability and generalizability of the primary findings.

### 3.3 ROC curves of PNI and SII for predicting 28-day and 90-day mortality in patients with different glucose metabolism status

**Figure 4:**
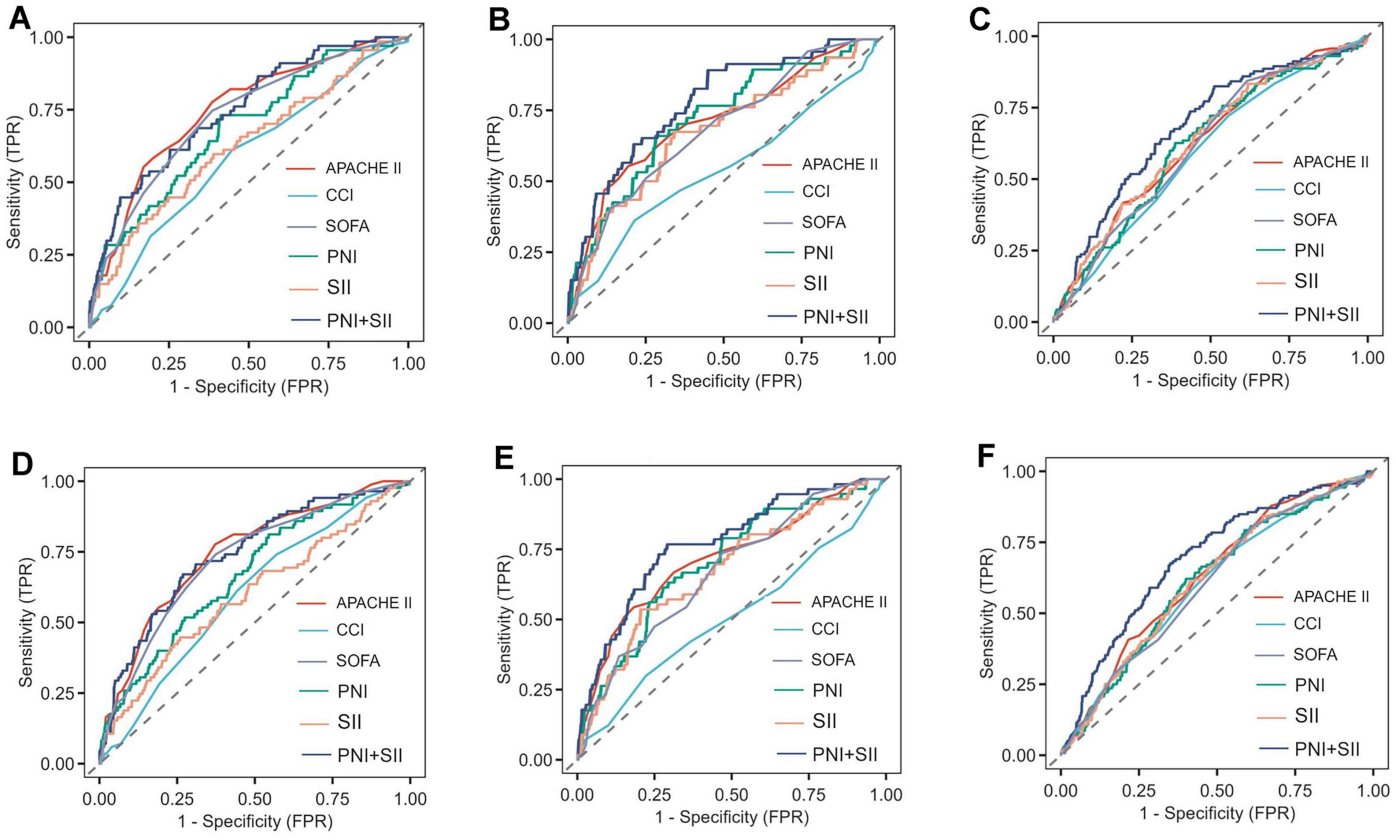
The ROC curves of PNI and SII as biomarkers for predicting 28-day mortality **(A–C)** and 90-day mortality **(D–F)**. **(A)** PNI versus SII versus PNI + SII versus APACHE II, CCI, and SOFA in NGR patients for predicting 28-day mortality. **(B)** PNI versus SII versus PNI + SII versus APACHE II, CCI, and SOFA in pre-DM patients for predicting 28-day mortality. **(C)** PNI versus SII versus PNI + SII versus APACHE II, CCI, and SOFA in DM patients for predicting 28-day mortality. **(D)** PNI versus SII versus PNI + SII versus APACHE II, CCI, and SOFA in NGR patients for predicting 90-day mortality. **(E)** PNI versus SII versus PNI + SII versus APACHE II, CCI, and SOFA in pre-DM patients for predicting 90-day mortality. **(F)** PNI versus SII versus PNI + S versus APACHE II, CCI, and GCS in DM patients for predicting 90-day mortality.

Predictive analyses of mortality in critically ill patients with cardiovascular and cerebrovascular diseases across different glucose metabolic statuses, based on ROC curves and model performance comparison tables, revealed the following (Supplementary Table S3): For 28-day mortality prediction: In the NGR subgroup (Figure 4A), the PNI + SII combined model yielded an AUC of 0.746 (95%CI: 0.684–0.808), which was comparable to that of the APACHE II score (0.747, *p* = 0.971) but with higher specificity (0.747 vs. 0.615), rendering it suitable for ruling out low-risk patients. In the Pre-DM subgroup (Figure 4B), the combined model exhibited optimal performance (AUC = 0.775), significantly outperforming APACHE II (0.710, *p* = 0.041) and single indices (PNI/SII: AUC = 0.717/0.671). With a sensitivity of 0.891, it highlighted a strong capacity for early warning of high-risk patients. In the DM subgroup (Figure 4C), the AUC of the combined model decreased to 0.683; although it outperformed SII (0.632, *p* = 0.006) and CCI (0.593, *p* = 0.020), there was an imbalance between sensitivity (0.825) and specificity (0.489), suggesting that diabetic metabolic disturbances impaired its predictive efficacy. A similar trend was observed for 90-day mortality prediction: In the NGR subgroup (Figure 4D), the combined model had an AUC of 0.741, which remained comparable to that of APACHE II (0.747). In the Pre-DM subgroup (Figure 4E), the combined model maintained optimal performance (AUC = 0.770) with balanced sensitivity (0.857) and specificity (0.709). In the DM subgroup (Figure 4F), the combined model achieved an AUC of 0.694; while it outperformed single indices (e.g., SII: AUC = 0.617, *p* < 0.001), its ROC curve was closer to the diagonal.

Consistent with these discrimination results, Decision Curve Analysis showed that the PNI + SII combined model provided a higher net clinical benefit across clinically relevant threshold probabilities in the NGR and Pre-DM subgroups, whereas its net benefit was attenuated in the DM subgroup (Supplementary Figure S1).

In summary, the following conclusions can be drawn: (1) The PNI + SII combined model exhibited excellent predictive performance in non-diabetic (NGR and Pre-DM) patients, with the highest AUC observed in the Pre-DM subgroup. (2) In diabetic patients, metabolic disturbances (characterized by chronic inflammation and metabolic disorders) led to reduced sensitivity and low specificity of the combined model, resulting in significantly weaker predictive efficacy compared to non-diabetic subgroups. (3) The model demonstrated strong predictive power for short-term (28-day) and medium-term (90-day) mortality risks in Pre-DM patients (AUC > 0.77, sensitivity>0.85), making it a valuable tool for early intervention.

### 3.4 Machine learning of PNI and SII to predict 28-day and 90-day mortality in patients with different glucose metabolism status

#### 3.4.1 Algorithm of Boruta

**Figure 5:**
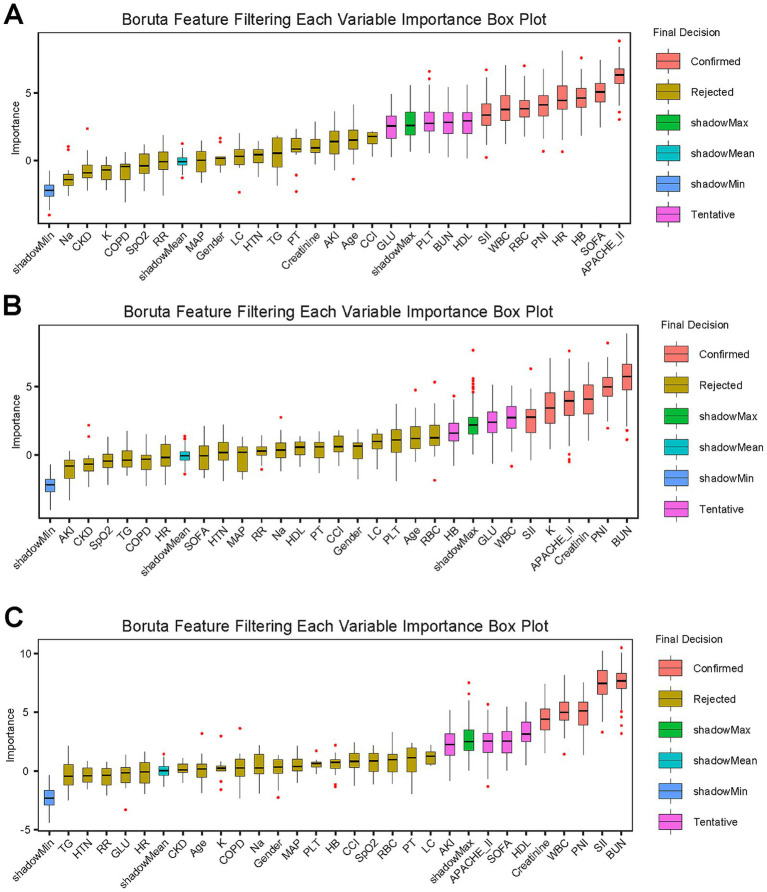
The Boruta algorithm ranks the importance of potential risk factors for 28-day mortality. The *x*-axis delineates parameter nomenclature, whereas the *y*-axis quantifies standardized scores (Z-scores) across variables. Boxplot distributions graphically depict the dispersion characteristics of normalized values during model computation cycles. **(A)** Patients with NGR; **(B)** patients with pre-DM; **(C)** patients with DM.

The final variables incorporated into the machine learning models were derived from feature selection via the Boruta algorithm, which aimed to identify the most predictive features for “all-cause mortality in critically ill patients with cardiovascular and cerebrovascular diseases across different glucose metabolic states” from a multitude of clinical indicators. Results of the Boruta algorithm in the overall population (Figure 5).

#### 3.4.2 Machine learning: PNI, SII, and 28-day mortality

**Figure 6:**
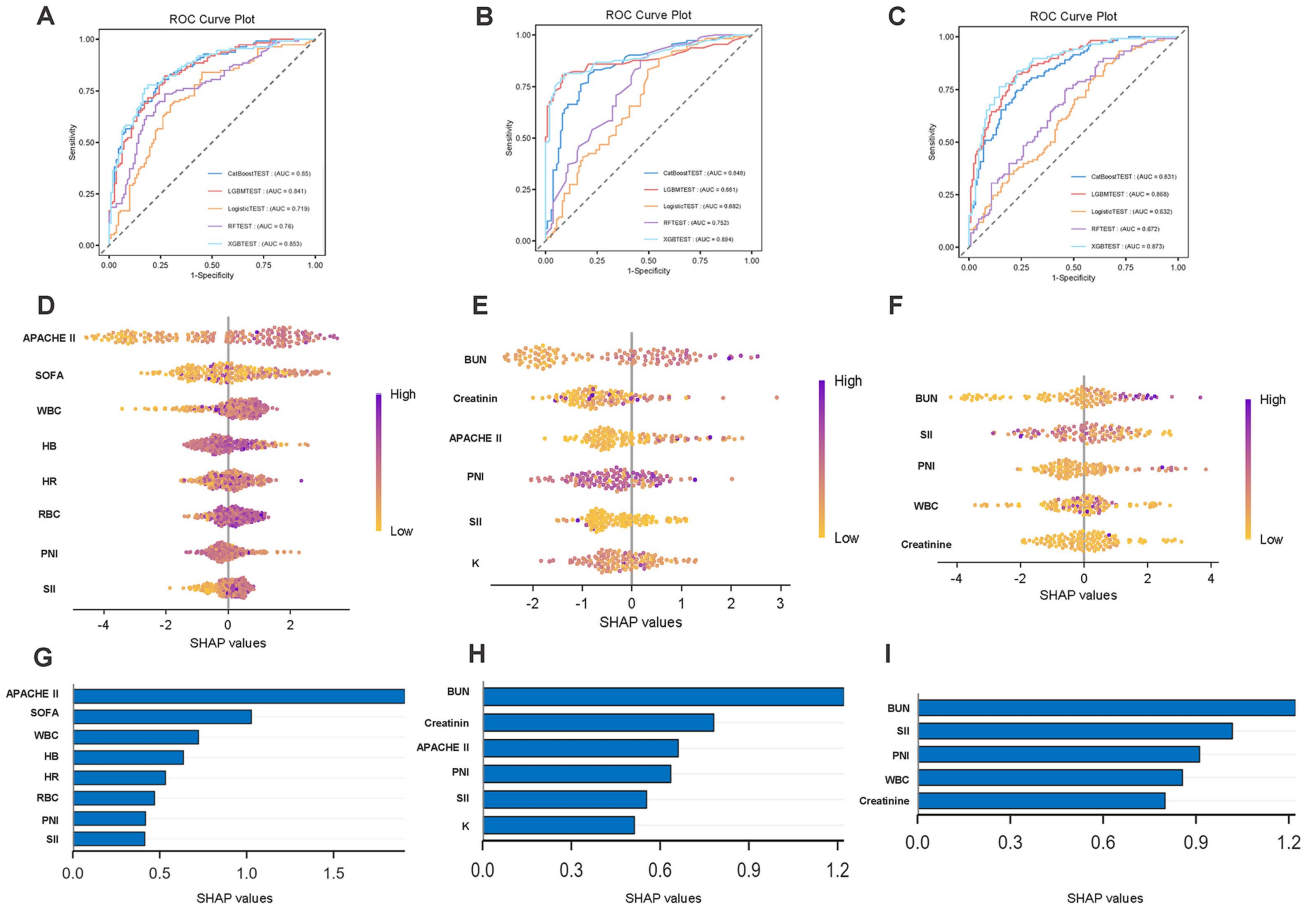
ROC curves and SHAP interpretation of ML-based 28-day mortality prediction models. **(A,D,G)** Patients with NGR; **(B,E,H)** patients with Pre-DM; **(C,F,I)** patients with DM. LR, logistic regression; CatBoost, categorical boosting; RF, random forest; XGBoost, extreme gradient boosting; LightGBM, light gradient boosting machine.

This study utilized machine learning to dissect the prognostic value of PNI and SII for all-cause mortality in critically ill patients with cardiovascular and cerebrovascular diseases across different glucose metabolic states from three dimensions (Supplementary Table S9): The ROC curve analyses (Figures 6A–C), are as follows: 1. In the NGR population, XGBoost (AUC = 0.853) and CatBoost (AUC = 0.850) performed optimally, followed by LGBM (AUC = 0.841), while RF (0.760) and LR (0.719) performed less favorably. XGBoost exhibited superior metrics including sensitivity (0.754) and specificity (0.730). 2. In the Pre-DM population, XGBoost (AUC = 0.894) and LGBM (AUC = 0.881) were the top performers, outperforming CatBoost (0.848), RF (0.752), and LR (0.683) by a large margin. XGBoost showed notable sensitivity (0.916) and specificity (0.928), with its ROC curve far from the diagonal, indicating the highest efficiency in integrating PNI and SII. 3. In the DM population, LGBM (AUC = 0.868) ranked first, followed by XGBoost (0.823), while CatBoost (0.831), RF (0.672), and LR (0.632) performed less well. LGBM showed better performance in balancing risk identification and misclassification rates; however, its ROC curve was closer to the diagonal compared to those in the NGR and Pre-DM populations, consistent with the conclusion that diabetes mellitus impairs predictive efficacy. 2. SHAP illustrated the effects of PNI and SII (Figures 6D–F): For PNI, in the NGR group, low PNI values were concentrated in the positive SHAP value region, while high PNI values were mostly in the negative region, indicating that reduced PNI was clearly associated with increased mortality risk with a stable effect. In the Pre-DM group, low PNI still dominated positive SHAP values but with a scattered distribution, suggesting that the association between PNI and risk was slightly disturbed by metabolic disorders. In the DM group, the distribution of SHAP values for PNI was disorganized. For SII, in the NGR group, high SII values were concentrated in the positive SHAP value region, while low SII values were mostly in the negative region, with a clear effect of immune-inflammatory activation driving mortality risk. In the Pre-DM group, high SII still dominated positive SHAP values, but some low SII values showed positive values, indicating that impaired glucose tolerance began to weaken the predictive effect of SII. In the DM group, SHAP values for SII were mixed between positive and negative, with the positive value region contracted. 3. SHAP feature importance (Figures 6G–I) showed that in the NGR group, PNI and SII exhibited high absolute values of mean SHAP values and ranked high, serving as core predictors of mortality risk in patients with normal glucose metabolism. In the Pre-DM group, both maintained relatively high importance but with slightly lower rankings, as metabolic abnormalities began to dilute their effects. In the DM group, the bars for both were significantly shortened with declining rankings, indicating that the complex mechanisms of diabetes dominated risk, weakening their predictive value.

In summary, glucose metabolic status determines the predictive value of PNI + SII. In NGR/Pre-DM populations, low PNI and high SII are strong predictors of mortality risk, with high model prediction accuracy. In the DM population, due to associations with chronic pathological remodeling in diabetes, the predictive efficacy of both indices is significantly attenuated, requiring assessment in combination with more complex indicators. However, considering that mortality outcomes are inherently imbalanced, ROC curves alone may not fully characterize model performance. Therefore, confusion matrices of the internal testing set were additionally provided to display the distribution of true positives, false positives, true negatives, and false negatives, thereby facilitating a more comprehensive evaluation of classification behavior and model limitations (Supplementary Figure S2).

**Figure 7:**
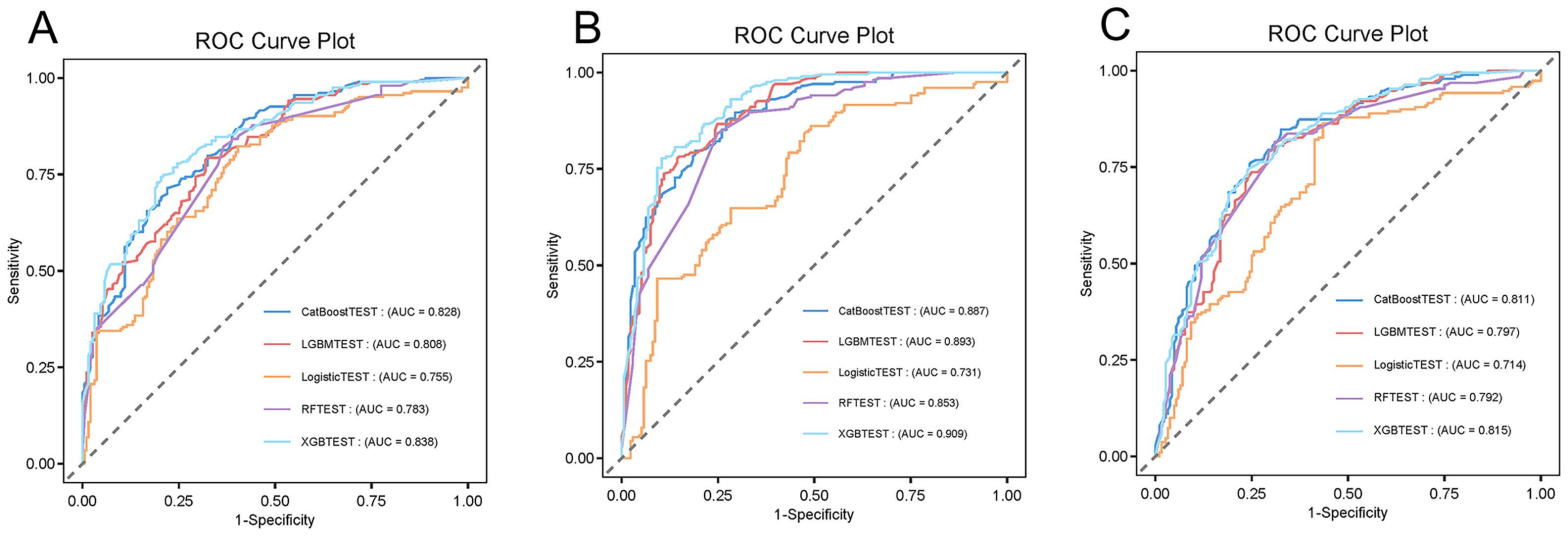
External validation of ML-based 28-day mortality prediction models using patient data from the Affiliated Hospital of Qingdao University and Qingdao Municipal Hospital. **(A)** Patients with NGR; **(B)** patients with Pre-DM; **(C)** patients with DM. The external validation results demonstrate consistent predictive trends with the training set, further supporting the robustness and generalizability of the model.

Additionally, patients from The Affiliated Hospital of Qingdao University and Qingdao Municipal Hospital were included as external validation cohorts for the machine learning models, with detailed cohort-related analyses provided in Supplementary Tables S10, S11 and Supplementary Figures S3, S4. The validation results demonstrated that predictive trends in the external datasets were consistent with those observed in the original training cohort, further supporting the robustness and generalizability of our findings (Figures 7A–C and Supplementary Figure S5).

## 4 Discussion

This study systematically explored the value of combined PNI and SII assessment in predicting all-cause mortality among critically ill patients with cardiovascular and cerebrovascular diseases across different glucose metabolic states. For the first time, it unraveled the prognostic significance of PNI and SII—both individually and in combination—via Kaplan–Meier curves, Cox proportional hazards regression, RCS analysis, and machine learning approaches. Key findings include the following: PNI is negatively correlated with mortality risk, while SII is positively correlated with mortality risk; the predictive performance of their combination is significantly superior to that of either index alone or traditional comorbidity scores, with particularly prominent efficacy in patients with Pre-DM. Additionally, this association is modulated by glucose metabolic status: for instance, the protective effect of PNI on 90-day mortality is abrogated in patients with DM, whereas the adverse effect of SII remains consistent across all metabolic states.

### 4.1 Mechanism of association between PNI, SII, and the prognosis of cardiovascular and cerebrovascular diseases

This study identified that decreased PNI and elevated SII are both independent risk factors for 28-day and 90-day all-cause mortality in critically ill patients with cardiovascular and cerebrovascular diseases, and their combined application markedly enhances predictive efficacy. This result aligns with conclusions from previous studies, further confirming the pivotal role of nutritional status and immune-inflammatory responses in disease prognosis.

In recent years, low serum albumin levels have increasingly been recognized as a risk factor and predictor of mortality across a spectrum of diseases—including cirrhosis, malnutrition, nephrotic syndrome, and sepsis—regardless of gender, age, comorbidities, or genetic polymorphisms (20, 21). Serum albumin plays a pivotal role in multiple physiological processes: maintaining plasma colloid osmotic pressure; binding and transporting various endogenous and exogenous ligands to target tissues or biotransformation sites; modulating enzyme activity to participate in pharmacokinetic and toxicokinetic processes; and regulating immune responses (22–25). A low PNI indicates insufficient protein intake or synthesis. As the fundamental building block of human cells and tissues, protein is critical for patients with severe cardiovascular and cerebrovascular diseases, particularly after hemorrhage: cardiovascular and cerebrovascular and neural tissues, which sustain varying degrees of damage, require adequate protein to support repair processes. Inadequate protein not only depletes essential raw materials but also slows down substance transport, thereby delaying the repair of damaged tissues. Beyond cardio-cerebral tissues, protein deficiency associated with low PNI can impair other systemic organs and tissues, leading to overall functional decline and restricted mobility. Prolonged immobility is recognized as a significant risk factor for venous thromboembolism and hypostatic pneumonia (26, 27). Additionally, peripheral blood lymphocytes—core components of the immune system—are vital for defending against pathogenic invasion and initiating immune responses (28, 29). Following the onset of cardiovascular and cerebrovascular cardiovascular and cerebrovascular diseases, patients often enter a state of physiological stress, rendering their bodies inherently susceptible to invasion by various pathogens. For instance, bacteria can infiltrate via the respiratory or urinary systems, triggering infections (30, 31). The immunocompromise reflected by low PNI, however, creates vulnerabilities in the body’s immune defense barrier. Reduced lymphocyte counts and impaired lymphocyte function hinder the effective recognition and clearance of foreign pathogens, as well as the timely initiation of robust immune responses—thereby elevating the risk of infections. Such infections, including pulmonary, intracranial, and urinary tract infections, are particularly prevalent in this patient population. Once an infection occurs, it further amplifies systemic inflammatory responses, exacerbating the already fragile physical state, depleting additional energy and nutrients, and establishing a vicious cycle. This ultimately leads to a significantly increased risk of death within 28 days. Normal immune regulatory mechanisms are critical for maintaining internal homeostasis in the context of disease. In patients with low PNI, excessive inflammatory responses may fail to be effectively regulated, leading to the release of large quantities of proinflammatory mediators such as interleukins and tumor necrosis factor (32, 33). These proinflammatory mediators not only exacerbate local ischemic damage to cardiac and cerebral tissues but also disrupt systemic circulatory and metabolic homeostasis, exerting adverse effects on vital organs including the kidneys and liver, thereby indirectly increasing the likelihood of patient mortality (34–36). Conversely, the body’s immune repair capacity is also impaired, hindering the repair process of damaged brain tissue and other tissues following hemorrhage, delaying recovery, further deteriorating prognosis, and elevating 28-day mortality.

Elevated SII, as a risk factor for mortality in patients with cardiovascular and cerebrovascular diseases, is mechanistically rooted in the synergistic interplay of immune-inflammatory imbalance, thrombotic propensity, and vascular injury. This can be elaborated as follows: A core component of high SII lies in elevated neutrophil counts. As the primary effector cells in acute inflammation, neutrophils, under pathological conditions, release substantial amounts of reactive oxygen species (ROS), neutrophil extracellular traps (NETs), and inflammatory cytokines (e.g., TNF-*α*, IL-6) (37, 38). These mediators directly damage vascular endothelial cells, disrupt the integrity of the endothelial barrier, promote lipid deposition and foam cell formation, and accelerate the initiation and progression of atherosclerotic plaques (39). Concurrently, excessive inflammatory responses amplify local tissue damage (such as myocardial ischemia–reperfusion injury and edema in cerebral infarct regions), exacerbating organ dysfunction—a critical driver of increased mortality risk following acute cardiovascular and cerebrovascular events (40, 41). Platelets constitute another pivotal component of SII; elevated platelet counts or hyperactive platelet function significantly heighten the risk of thrombosis. In states of elevated platelet activity, platelets exhibit increased adherence to damaged vascular endothelium, releasing procoagulant substances such as thromboxane A2 (TXA2) and adenosine diphosphate (ADP), which activate the coagulation cascade, promote fibrin deposition, and ultimately facilitate thrombus formation (42, 43). In coronary or cerebral arteries, such thrombi can directly occlude blood vessels, leading to acute myocardial infarction or ischemic stroke. Once an acute event occurs, the body—due to impaired immune function (lymphopenia)—exhibits reduced tolerance to ischemic–hypoxic insults, ultimately resulting in elevated mortality risk. Furthermore, RCS analysis revealed that in patients with DM, mortality risk rises precipitously when SII exceeds 10,000, displaying an “accelerating effect.” This suggests that intensified anti-inflammatory interventions are warranted within this range to halt the escalation of risk. In contrast, SII shows a linear association with mortality risk in patients with NGR, indicating that in the absence of underlying metabolic abnormalities, the impact of inflammation on prognosis is more dependent on cumulative effects. Thus, clinical interventions may prioritize sustained control over threshold-triggered measures.

The present study found that the predictive performance of the combined assessment of PNI and SII (AUC: 0.683–0.775) was significantly superior to that of either single indicator (PNI: 0.626–0.684; SII: 0.632–0.684). Notably, in the prediabetes (Pre-DM) subgroup, its predictive efficacy even surpassed that of the classical APACHE II score (AUC: 0.775 vs. 0.747). This result confirms the synergistic effect of the “nutrient-immune” network—while a single indicator can only reflect one dimension of the body’s pathophysiological state, combined assessment can more comprehensively capture the prognostic risk in critically ill patients with cardiovascular and cerebrovascular diseases, addressing the limitations of predictive bias associated with existing single indicators.

### 4.2 The regulatory effect of glucose metabolism status on the prediction performance of PNI and SII

The key findings of this study indicate that PNI and SII exhibit a significant dependence on metabolic status in terms of their predictive value. In NGR patients, the synergistic protective effect of high PNI (indicating adequate nutrition and intact immune function) and low SII (reflecting controlled inflammation) remains stable, as evidenced by distinct separation in Kaplan–Meier curves and consistent hazard ratios in Cox models. In Pre-DM patients, although this effect is slightly modified by mild metabolic disturbances (with a slight reduction in curve separation), the combined assessment still serves as the optimal predictor (AUC 0.775). However, in DM patients, the predictive efficacy of both indices is significantly attenuated. Meanwhile, the protective effect of PNI is also metabolically status-dependent: in 28-day mortality, the protective effect of PNI spans all metabolic states; notably, in 90-day mortality, this protective effect of PNI completely vanishes in DM patients. A potential mechanism is that insulin resistance in diabetic patients impairs cellular uptake and utilization of glucose, amino acids, and other nutrients, preventing tissues such as muscle and myocardium from effectively utilizing circulating nutrients, thereby limiting energy supply and protein synthesis (44). The 28-day mortality primarily reflects survival capacity during the acute critical phase. At this stage, the nutritional reserve (albumin levels) and immune function (lymphocyte count) represented by PNI directly influence the body’s tolerance to acute injury: adequate nutrition maintains cellular metabolism and organ function, while normal immune function inhibits the spread of infection, thereby reducing short-term mortality risk (45). Even in diabetic patients, those with higher PNI (indicating better nutritional and immune status) can still withstand acute insults in the short term; thus, the protective effect of PNI within 28 days extends across all metabolic states. In contrast, 90-day mortality more strongly reflects the cumulative impact of chronic pathological processes, with drivers shifting toward chronic complications (e.g., microangiopathy, cardiovascular events), persistent damage from metabolic disturbances (e.g., hyperglycemic toxicity), and long-term immune exhaustion. Here, the protective effect of isolated nutritional or immune reserve (PNI) may be overshadowed by more complex chronic pathological mechanisms, particularly in diabetic patients. Additionally, microvascular and macrovascular complications associated with diabetes often coexist with comorbidities such as hypertension, coronary heart disease, and renal insufficiency, which impair the function of organs including the heart, brain, and kidneys (46). These conditions may progress within 90 days (e.g., acute myocardial infarction, renal failure) and become primary causes of death. In contrast to PNI, SII— as a quantitative indicator of immune inflammation—exhibits a consistent positive correlation with mortality risk across all metabolic states, with an even stronger association in DM patients. This suggests that the chronic low-grade inflammation in DM patients synergizes with immune activation during the acute critical phase, further amplifying inflammatory damage to organ function.

### 4.3 The clinical advantages, application value and limitations of PNI combined with SII

This study ensured the robustness of its findings through rigorous statistical methodologies: Stratified analyses using Cox proportional hazards models effectively controlled for confounding effects of key factors (such as age, AKI, and hemoglobin). The Schoenfeld residual test validated the temporal stability of hazard ratios (with *p* > 0.05 in the NGR and Pre-DM groups). RCS curves revealed non-linear relationships between PNI/SII and mortality (e.g., the protective effect of PNI exhibits a diminishing trend). Additionally, ROC curves and AUC comparisons quantitatively demonstrated the superiority of predictive performance. The integration of machine learning techniques further enhanced the depth and interpretability of the results: The Boruta algorithm confirmed the core predictive value of PNI and SII in the NGR and Pre-DM groups. SHAP analyses intuitively illustrated the effect directions of these indices on mortality risk (i.e., lower PNI → increased risk; higher SII → increased risk) and the dependence of their effect strengths on metabolic status. This combination of “traditional statistics and machine learning” not only verified the statistical significance of the combined assessment but also uncovered its intrinsic operational patterns, laying a solid foundation for the clinical translation of the results. This study has several limitations: First, the retrospective design may introduce selection bias. Although the MIMIC-IV database has a large sample size and standardized de-identification procedures, the single-center nature of the data may restrict the generalizability of the findings, warranting validation in future multicenter prospective studies. Second, PNI and SII were calculated solely based on baseline values, without incorporating dynamic changes; their fluctuations during hospitalization may better reflect disease progression. Third, the classification of glucose metabolic status relied on HbA1c and medical history, failing to completely exclude the interference of stress-induced hyperglycemia. Despite reducing bias through repeated blood glucose measurements, more stringent metabolic assessment criteria are needed for further optimization.

## Data Availability

The raw data supporting the conclusions of this article will be made available by the authors, without undue reservation.
