# Supplementary material for: Multicenter evaluation of prognostic nutritional index and systemic immune-inflammation index in predicting mortality among critically ill cardiovascular and cerebrovascular patients with varied glucose metabolism: a machine learning-based cohort study
Source: Front Nutr. 2026 Feb 3;13:1703589. doi: 10.3389/fnut.2026.1703589 (PMC12909231; doi:10.3389/fnut.2026.1703589)
Supplement: Supplementary file 1 [file Table_1.docx]

Supplementary materials

**Table S1 Disease codes included in the study**

| Ischemic | ICD9 | 433,43301,4331,43311,4332,43321,4333,43331,4 |
| --- | --- | --- |
| stroke |  | 338,43381,4339,43391,434,43401,4341,43411,43 |
|  |  | 49,43491 |
|  | ICD10 | I6341, I6342, I6343, I6344, I635, I6351, I63519, I6352, |
|  |  | I6353, I6354, |
|  |  | I65, I66, I6340, I63411, I63412, I63413, I63419, I63421, |
|  |  | I63422, I63423, |
|  |  | I63429, I63431, I63432, I63433, I63439, I63441, |
|  |  | I63442, I63443, I63449, |
|  |  | I639, I6350, I63511, I63512, I63513, I63521, I63522, |
|  |  | I6.523, I63529, |
|  |  | I63531, I63532, I63533, I63539, I63541, I63542, |
|  |  | I63543, I63549, I639, |
|  |  | I636, I638, I6381, I6389, I639 |
| Hemorrhagic | ICD9 | 430,431,4320,4321,4329 |
| stroke | ICD10 | I60, I6001, I6002, I6010, I6011, I6012. I602, I6020, I6021 |
|  |  | I6022, I6031, I6032, I604, I6051, I6052, I606. I607, I608 |
|  |  | I609, I610, I611, I612, I613, I614, I615, I616, I618, I619 |
|  |  | I6200, I6201, I6202, I6203, I621, I629 |
| Ischemic | ICD10 | I25.5, I20.0, I20.1, I20.8, I20.9, I21.0, I21.01, I21.02, I21.09, |
| heart disease |  | I21.11, I21.19, |
|  |  | I21.21, I21.29, I21.3, I21.4, I21.9, I21.A1, I21.A9, I22.0, |
|  |  | I22.1, I22.2, I22.8, |
|  |  | I22.9, I23.0, I23.1, I23.2, I23.3, I23.4, I23.5, I23.6, I23.7, |
|  |  | I23.8, I24.0, I24.1, |
|  |  | I24.8, I24.9, I25.10, I25.110, I25.111, I25.118, I25.119, |
|  |  | I25.810, I25.82, I25.83, |

**Table S2 The association of the PNI and SII with 28-day and 90-day mortality**

| **Variables** | **Model 1** | | | |  | | **Model 2** | |  | |  | **Model 3** | | | |
| --- | --- | --- | --- | --- | --- | --- | --- | --- | --- | --- | --- | --- | --- | --- | --- |
|  | **HR (95%CI)** | | **P** | |  | | **HR (95%CI)** | | **P** | |  | **HR (95%CI)** | | **P** | |
| **28-day mortality** |  | |  | |  | |  | |  | |  |  | |  | |
| **Overall** |  | |  | |  | |  | |  | |  |  | |  | |
| PNI |  | |  | |  | |  | |  | |  |  | |  | |
| T1 | 1.00 (Reference) | |  | |  | | 1.00 (Reference) | |  | |  | 1.00 (Reference) | |  | |
| T2 | 0.574 (0.425 ~ 0.776) | | <0.001 | |  | | 0.569 (0.421 ~ 0.769) | | <0.001 | |  | 0.713 (0.524 ~ 0.970) | | 0.031 | |
| T3 | 0.395 (0.281 ~ 0.555) | | <0.001 | |  | | 0.405 (0.288 ~ 0.569) | | <0.001 | |  | 0.582 (0.404 ~ 0.838) | | 0.004 | |
| P for trend | 0.620 (0.525 ~ 0.733) | | <0.001 | |  | | 0.626 (0.529 ~ 0.740) | | <0.001 | |  | 0.755 (0.632 ~ 0.902) | | 0.002 | |
| SII |  | |  | |  | |  | |  | |  |  | |  | |
| T1 | 1.00 (Reference) | |  | |  | | 1.00 (Reference) | |  | |  | 1.00 (Reference) | |  | |
| T2 | 1.222 (0.847 ~ 1.764) | | 0.284 | |  | | 1.251 (0.866 ~ 1.806) | | 0.233 | |  | 1.126 (0.774 ~ 1.638) | | 0.535 | |
| T3 | 2.360 (1.70 ~ 3.276) | | <0.001 | |  | | 2.394 (1.724 ~ 3.324) | | <0.001 | |  | 2.072 (1.486 ~ 2.888) | | <0.001 | |
| P for trend | 1.582 (1.339 ~ 1.868) | | <0.001 | |  | | 1.590 (1.347 ~ 1.877) | | <0.001 | |  | 1.488 (1.258~ 1.761) | | <0.001 | |
| **Patients with NGR** | | |  | |  | |  | |  | |  |  | |  | |
| PNI |  | |  | |  | |  | |  | |  |  | |  | |
| T1 | 1.00 (Reference) | |  | |  | | 1.00 (Reference) | |  | |  | 1.00 (Reference) | |  | |
| T2 | 0.457 (0.265 ~ 0.790) | | 0.005 | |  | | 0.445 (0.257 ~ 0.770) | | 0.004 | |  | 0.522 (0.300 ~ 0.908) | | 0.021 | |
| T3 | 0.251 (0.128 ~ 0.494) | | <0.001 | |  | | 0.259 (0.131 ~ 0.509) | | <0.001 | |  | 0.350 (0.175 ~ 0.699) | | 0.003 | |
| P for trend | 0.492 (0.356 ~ 0.680) | | <0.001 | |  | | 0.495 (0.356 ~ 0.686) | | <0.001 | |  | 0.576 ( 0.412~ 0.805) | | <0.001 | |
| SII |  | |  | |  | |  | |  | |  |  | |  | |
| T1 | 1.00 (Reference) | |  | |  | | 1.00 (Reference) | |  | |  | 1.00 (Reference) | |  | |
| T2 | 0.789 (0.392 ~ 1.586) | | 0.506 | |  | | 0.803 (0.399 ~ 1.616) | | 0.538 | |  | 0.688(0.337 ~ 1.406) | | 0.305 | |
| T3 | 2.089 (1.183 ~ 3.689) | | 0.011 | |  | | 2.103 (1.191 ~ 3.713) | | 0.010 | |  | 1.905 (1.054 ~ 3.442) | | 0.033 | |
| P for trend | 1.536 (1.131 ~ 2.084) | | 0.006 | |  | | 1.538 (1.134 ~ 2.086) | | 0.006 | |  | 1.487 (1.081 ~ 2.045) | | 0.015 | |
| **Patients with Pre-DM** | | |  | |  | |  | |  | |  |  | |  | |
| PNI |  | |  | |  | |  | |  | |  |  | |  | |
| T1 | 1.00 (Reference) | |  | |  | | 1.00 (Reference) | |  | |  | 1.00 (Reference) | |  | |
| T2 | 0.540 (0.278 ~ 1.051) | | 0.07 | |  | | 0.560 (0.284 ~ 1.103) | | 0.094 | |  | 0.835 (0.416 ~ 1.677) | | 0.612 | |
| T3 | 0.318 (0.154 ~ 0.655) | | 0.002 | |  | | 0.318 (0.154 ~ 0.655) | | 0.002 | |  | 0.472 (0.214 ~ 0.945) | | 0.032 | |
| P for trend | 0.562 (0.392 ~ 0.806) | | 0.002 | |  | | 0.564 (0.393 ~ 0.807) | | 0.002 | |  | 0.699 (0.477 ~ 0.909) | | 0.031 | |
| SII |  | |  | |  | |  | |  | |  |  | |  | |
| T1 | 1.00 (Reference) | |  | |  | | 1.00 (Reference) | |  | |  | 1.00 (Reference) | |  | |
| T2 | 1.177 (0.553 ~ 2.503) | | 0.673 | |  | | 1.206 (0.566 ~ 2.573) | | 0.627 | |  | 1.197 (0.549 ~ 2.606) | | 0.651 | |
| T3 | 2.004 (0.997 ~ 4.029) | | 0.051 | |  | | 2.045 (1.012 ~ 4.133) | | 0.046 | |  | 1.971 (0.950 ~ 4.088) | | 0.068 | |
| P for trend | 1.435 (1.004 ~ 2.050) | | 0.048 | |  | | 1.448 (1.011 ~ 2.073) | | 0.043 | |  | 1.423 (0.982 ~ 2.061) | | 0.062 | |
| **Patients with DM** |  | |  | |  | |  | |  | |  |  | |  | |
| PNI |  | |  | |  | |  | |  | |  |  | |  | |
| T1 | 1.00 (Reference) | |  | |  | | 1.00 (Reference) | |  | |  | 1.00 (Reference) | |  | |
| T2 | 0.686 (0.445 ~ 1.059) | | 0.089 | |  | | 0.710 (0.460 ~ 1.096) | | 0.122 | |  | 0.887 (0.568 ~ 1.386) | | 0.599 | |
| T3 | 0.591 (0.368 ~ 0.948) | | 0.029 | |  | | 0.622 (0.386 ~ 0.952) | | 0.036 | |  | 0.957 (0.573 ~ 0.987) | | 0.043 | |
| P for trend | 0.756 (0.599 ~ 0.956) | | 0.019 | |  | | 0.777 (0.614 ~ 0.982) | | 0.035 | |  | 0.858 (0.649 ~ 0.992) | | 0.042 | |
| SII | |  | |  | |  | |  | |  | | |  | |  |
| T1 | | 1.00 (Reference) | |  | | 1.00 (Reference) | |  | | 1.00 (Reference) | | |  | |  |
| T2 | | 1.609 (0.937 ~ 2.764) | | 0.085 | | 1.664 (0.968 ~ 2.860) | | 0.065 | | 1.407 (0.810 ~ 2.446) | | | 0.226 | |  |
| T3 | | 2.770 (1.683 ~ 4.559) | | <0.001 | | 2.809 (1.706 ~ 4.627) | | <0.001 | | 2.126 (1.279 ~ 3.534) | | | 0.004 | |  |
| P for trend | | 1.674 (1.317 ~ 2.129) | | <0.001 | | 1.678 (1.321 ~ 2.132) | | <0.001 | | 1.467 (1.149 ~ 1.873) | | | 0.002 | |  |
| **90-day mortality** | |  | |  | |  | |  | |  | | |  | |  |
| **Overall** | |  | |  | |  | |  | |  | | |  | |  |
| PNI | |  | |  | |  | |  | |  | | |  | |  |
| T1 | | 1.00 (Reference) | |  | | 1.00 (Reference) | |  | | 1.00 (Reference) | | |  | |  |
| T2 | | 0.783 (0.597 ~ 1.026) | | 0.076 | | 0.789 (0.601 ~ 1.034) | | 0.086 | | 0.755 (0.572 ~ 0.995) | | | 0.046 | |  |
| T3 | | 0.666 (0.486 ~ 0.912) | | 0.011 | | 0.691 (0.504 ~ 0.947) | | 0.021 | | 0.590 (0.423 ~ 0.823) | | | 0.002 | |  |
| P for trend | | 0.811 (0.696 ~ 0.945) | | 0.007 | | 0.824 (0.707 ~ 0.961) | | 0.013 | | 0.766 (0.652 ~ 0.900) | | | 0.001 | |  |
| SII | |  | |  | |  | |  | |  | | |  | |  |
| T1 | | 1.00 (Reference) | |  | | 1.00 (Reference) | |  | | 1.00 (Reference) | | |  | |  |
| T2 | | 1.205 (0.871 ~ 1.666) | | 0.260 | | 1.251 (0.904 ~ 1.731) | | 0.176 | | 1.298 (0.932 ~ 1.808) | | | 0.123 | |  |
| T3 | | 1.842 (1.366 ~ 2.483) | | <0.001 | | 1.861 (1.379 ~ 2.510) | | <0.001 | | 1.925 (1.421 ~ 2.607) | | | <0.001 | |  |
| P for trend | | 1.377 (1.186 ~ 1.599) | | <0.001 | | 1.484 (1.134 ~ 1.944) | | 0.004 | | 1.399 (1.204 ~ 1.625) | | | <0.001 | |  |
| **Patients with NGR** | | | |  | |  | |  | |  | | |  | |  |
| PNI | |  | |  | |  | |  | |  | | |  | |  |
| T1 | | 1.00 (Reference) | |  | | 1.00 (Reference) | |  | | 1.00 (Reference) | | |  | |  |
| T2 | | 0.673 (0.416 ~ 1.088) | | 0.106 | | 0.666 (0.412 ~ 1.079) | | 0.099 | | 0.606 (0.373 ~ 0.985) | | | 0.043 | |  |
| T3 | | 0.482 (0.263 ~ 0.885) | | 0.019 | | 0.510 (0.277 ~ 0.939) | | 0.031 | | 0.415 (0.223 ~ 0.773) | | | 0.006 | |  |
| P for trend | | 0.690 (0.517 ~ 0.921) | | 0.012 | | 0.704 (0.526 ~ 0.943) | | 0.019 | | 0.636 (0.472 ~ 0.856) | | | 0.003 | |  |
| SII | |  | |  | |  | |  | |  | | |  | |  |
| T1 | | 1.00 (Reference) | |  | | 1.00 (Reference) | |  | | 1.00 (Reference) | | |  | |  |
| T2 | | 0.878 (0.487 ~ 1.581) | | 0.664 | | 0.914 (0.506 ~ 1.648) | | 0.764 | | 0.844 (0.463 ~ 1.538) | | | 0.580 | |  |
| T3 | | 2.049 (1.232 ~ 3.410) | | 0.006 | | 2.045 (1.229 ~ 3.402) | | 0.006 | | 1.850 (1.098 ~ 3.118) | | | 0.021 | |  |
| P for trend | | 1.492 (1.137 ~ 1.958) | | 0.004 | | 1.484 (1.134 ~ 1.944) | | 0.004 | | 1.418 (1.076 ~ 1.868) | | | 0.013 | |  |
| **Patients with Pre-DM** | | | |  | |  | |  | |  | | |  | |  |
| PNI | |  | |  | |  | |  | |  | | |  | |  |
| T1 | | 1.00 (Reference) | |  | | 1.00 (Reference) | |  | | 1.00 (Reference) | | |  | |  |
| T2 | | 0.700 (0.385 ~ 1.275) | | 0.244 | | 0.707 (0.386 ~ 1.294) | | 0.261 | | 0.774 (0.413 ~ 1.450) | | | 0.424 | |  |
| T3 | | 0.473 (0.239 ~ 0.936) | | 0.032 | | 0.468 (0.236 ~ 0.928) | | 0.030 | | 0.423 (0.202 ~ 0.886) | | | 0.023 | |  |
| P for trend | | 0.689 (0.493 ~ 0.963) | | 0.029 | | 0.686 (0.491 ~ 0.960) | | 0.028 | | 0.662 (0.465 ~ 0.944) | | | 0.023 | |  |
| SII | |  | |  | |  | |  | |  | | |  | |  |
| T1 | | 1.00 (Reference) | |  | | 1.00 (Reference) | |  | | 1.00 (Reference) | | |  | |  |
| T2 | | 1.295 (0.643 ~ 2.607) | | 0.469 | | 1.337 (0.662 ~ 2.698) | | 0.418 | | 1.517 (0.732 ~ 3.144) | | | 0.262 | |  |
| T3 | | 1.914 (0.994 ~ 3.686) | | 0.052 | | 1.961 (1.015 ~ 3.792) | | 0.045 | | 2.415 (1.204 ~ 4.844) | | | 0.013 | |  |
| P for trend | | 1.392 (1.004 ~ 1.931) | | 0.047 | | 1.407 (1.014 ~ 1.952) | | 0.041 | | 1.558 (1.106 ~ 2.196) | | | 0.011 | |  |
| **Patients with DM** | |  | |  | |  | |  | |  | | |  | |  |
| PNI | |  | |  | |  | |  | |  | | |  | |  |
| T1 | | 1.00 (Reference) | |  | | 1.00 (Reference) | |  | | 1.00 (Reference) | | |  | |  |
| T2 | | 0.864 (0.580 ~ 1.289) | | 0.475 | | 0.920 (0.617 ~ 1.374) | | 0.685 | | 0.901 (0.597 ~ 1.359) | | | 0.618 | |  |
| T3 | | 0.925 (0.595 ~ 1.440) | | 0.731 | | 1.019 (0.654 ~ 1.589) | | 0.934 | | 0.909 (0.569 ~ 1.453) | | | 0.691 | |  |
| P for trend | | 0.946 (0.762 ~ 1.176) | | 0.619 | | 0.996 (0.801 ~ 1.237) | | 0.968 | | 0.946 (0.752~ 1.189) | | | 0.633 | |  |
| SII | |  | |  | |  | |  | |  | | |  | |  |
| T1 | | 1.00 (Reference) | |  | | 1.00 (Reference) | |  | | 1.00 (Reference) | | |  | |  |
| T2 | | 1.402 (0.868 ~ 2.265) | | 0.167 | | 1.485 (0.917 ~ 2.404) | | 0.108 | | 1.571 (0.960 ~ 2.569) | | | 0.072 | |  |
| T3 | | 1.726 (1.095 ~ 2.719) | | 0.019 | | 1.753 (1.110 ~ 2.768) | | 0.016 | | 1.830 (1.151 ~ 2.910) | | | 0.011 | |  |
| P for trend | | 1.300 (1.047 ~ 1.614) | | 0.018 | | 1.300 (1.048 ~ 1.613) | | 0.017 | | 1.321 (1.063 ~ 1.641) | | | 0.012 | |  |

Model 1: unadjusted;

Model 2: adjusted for age and sex

Model 3: adjusted for age, sex, HTN, AKI, LC, HB, RBC, WBC, PT, Creatinine and BUN

**Table S3 Discrimination of each predictive model for outcomes**

| **Models** | **AUC (95% CI)** | **Sensitivity** | **Specificity** | **P** |
| --- | --- | --- | --- | --- |
| **28-day mortality** |  |  |  |  |
| **NGR** |  |  |  |  |
| PNI+SII | 0.746(0.684-0.808) | 0.612 | 0.747 | 1.00 (Reference) |
| PNI | 0.684 (0.616-0.752) | 0.731 | 0.588 | 0.006 |
| SII | 0.626(0.551-0.700) | 0.597 | 0.611 | 0.002 |
| APACHE II | 0.747(0.685-0.809) | 0.776 | 0.615 | 0.971 |
| CCI | 0.584(0.512-0.657) | 0.612 | 0.552 | 0.002 |
| SOFA | 0.726(0.661-0.790) | 0.746 | 0.615 | 0.591 |
| **Pre-DM** |  |  |  |  |
| PNI+SII | 0.775(0.704-0.847) | 0.891 | 0.552 | 1.00 (Reference) |
| PNI | 0.717(0.637-0.798) | 0.660 | 0.719 | 0.043 |
| SII | 0.671(0.583-0.760) | 0.674 | 0.659 | 0.020 |
| APACHE II | 0.710(0.625-0.796) | 0.553 | 0.806 | 0.041 |
| CCI | 0.540(0.441-0.639) | 0.362 | 0.786 | <0.001 |
| SOFA | 0.682(0.600-0.764) | 0.404 | 0.863 | 0.037 |
| **DM** |  |  |  |  |
| PNI+SII | 0.683(0.630-0.736) | 0.825 | 0.489 | 1.00 (Reference) |
| PNI | 0.619(0.564-0.674) | 0.626 | 0.613 | 0.105 |
| SII | 0.632(0.576-0.687) | 0.886 | 0.382 | 0.006 |
| APACHE II | 0.629(0.576-0.684) | 0.417 | 0.778 | 0.176 |
| CCI | 0.593(0.539-0.647) | 0.722 | 0.443 | 0.020 |
| SOFA | 0.619(0.566-0.672) | 0.844 | 0.384 | 0.095 |
| **90-day mortality** |  |  |  |  |
| **NGR** |  |  |  |  |
| PNI+SII | 0.741(0.684-0.799) | 0.671 | 0.732 | 1.00 (Reference) |
| PNI | 0.670(0.608-0.731) | 0.812 | 0.394 | <0.001 |
| SII | 0.599(0.530-0.667) | 0.447 | 0.741 | <0.001 |
| APACHE II | 0.747(0.691-0.802) | 0.777 | 0.628 | 0.881 |
| CCI | 0.598(0.537-0.660) | 0.741 | 0.430 | <0.001 |
| SOFA | 0.723(0.666-0.781) | 0.741 | 0.626 | 0.634 |
| **Pre-DM** |  |  |  |  |
| PNI+SII | 0.770(0.702-0.839) | 0.857 | 0.709 | 1.00 (Reference) |
| PNI | 0.702(0.628-0.776) | 0.632 | 0.706 | 0.042 |
| SII | 0.674(0.595-0.753) | 0.536 | 0.796 | 0.016 |
| APACHE II | 0.715(0.636-0.794) | 0.544 | 0.817 | 0.048 |
| CCI | 0.508(0.419-0.597) | 0.298 | 0.779 | <0.001 |
| SOFA | 0.670(0.596-0.745) | 0.737 | 0.529 | 0.032 |
| **DM** |  |  |  |  |
| PNI+SII | 0.694(0.646-0.742) | 0.676 | 0.650 | 1.00 (Reference) |
| PNI | 0.615(0.565-0.666) | 0.621 | 0.600 | 0.024 |
| SII | 0.617(0.567-0.667) | 0.612 | 0.588 | <0.001 |
| APACHE II | 0.634(0.585-0.683) | 0.879 | 0.327 | 0.091 |
| CCI | 0.608(0.557-0.659) | 0.721 | 0.450 | 0.019 |
| SOFA | 0.605(0.555-0.655) | 0.807 | 0.385 | 0.014 |

APACHE II, Acute Physiology and Chronic Health Evaluation II; SOFA, Sequential Organ Failure Assessment; CCI, Charlson Comorbidity Index; PNI, Prognostic nutritional index; SII, Systemic immune inflammation index

**Table S4 Results of the proportional hazards test (Cox model) in the NGR population**

| **Variable Name** | **P** |
| --- | --- |
| Age | 0.8737 |
| Gender | 0.1338 |
| HTN | 0.1886 |
| AKI | 0.7278 |
| LC | 0.6228 |
| HB | 0.0008 |
| RBC | 0.0019 |
| WBC | 0.7846 |
| PT | 0.4323 |
| Creatinine | 0.8123 |
| BUN | 0.8910 |
| Group | 0.3582 |
| GLOBAL | 0.0889 |

**Table S5 Results of the proportional hazards test (Cox model) in the Pre-DM population**

| **Variable Name** | **P** |
| --- | --- |
| Age | 0.6275 |
| Gender | 0.3838 |
| HTN | 0.3406 |
| AKI | 0.1700 |
| LC | 0.6086 |
| HB | 0.0019 |
| RBC | 0.0139 |
| WBC | 0.4012 |
| PT | 0.5261 |
| Creatinine | 0.5358 |
| BUN | 0.9748 |
| Group | 0.1738 |
| GLOBAL | 0.2109 |

**Table S6 Results of the proportional hazards test (Cox model) in the DM population**

| **Variable Name** | **p** |
| --- | --- |
| Age | 0.9133 |
| Gender | 0.7947 |
| HTN | 0.2030 |
| AKI | 0.0042 |
| LC | 0.3206 |
| HB | 0.3447 |
| RBC | 0.1780 |
| WBC | 0.2583 |
| PT | 0.3858 |
| Creatinine | 0.3841 |
| BUN | 0.7543 |
| Group | 0.0989 |
| GLOBAL | 0.0255 |

**Table S7 The association of the combination of PNI and SII with all-cause mortality after excluding individuals with any missing value**

| **Variables** | **Model 1** | | **Model 2** |  | **Model 3** | |
| --- | --- | --- | --- | --- | --- | --- |
|  | **HR (95%CI)** | **P** | **HR (95%CI)** | **P** | **HR (95%CI)** | **P** |
| **28-day mortality** |  |  |  |  |  |  |
| **Overall** |  |  |  |  |  |  |
| Group 1 | 1.00 (Reference) |  | 1.00 (Reference) |  | 1.00 (Reference) |  |
| Group 2 | 1.720 (1.150 ~ 2.580) | 0.008 | 1.680 (1.120 ~ 2.520) | 0.012 | 1.550 (1.020 ~ 2.350) | 0.040 |
| Group 3 | 2.450 (1.300 ~ 4.620) | 0.006 | 2.510 (1.330 ~ 4.740) | 0.004 | 2.380 (1.250 ~ 4.530) | 0.008 |
| Group 4 | 3.050 (2.000 ~ 4.650) | <0.001 | 2.980 (1.950 ~ 4.550) | <0.001 | 2.620 (1.700 ~ 4.040) | <0.001 |
| P for trend | 1.380 (1.240 ~ 1.540) | <0.001 | 1.370 (1.230 ~ 1.530) | <0.001 | 1.310 (1.170 ~ 1.460) | <0.001 |
| **Patients with NGR** | |  |  |  |  |  |
| Group 1 | 1.00 (Reference) |  | 1.00 (Reference) |  | 1.00 (Reference) |  |
| Group 2 | 2.550 (1.100 ~ 5.910) | 0.029 | 2.480 (1.070 ~ 5.750) | 0.034 | 2.220 (0.950 ~ 5.190) | 0.048 |
| Group 3 | 2.520 (0.740 ~ 8.590) | 0.138 | 2.650 (0.780 ~ 9.010) | 0.120 | 2.780 (0.800 ~ 9.660) | 0.110 |
| Group 4 | 4.850 (2.100 ~ 11.200) | <0.001 | 4.680 (2.020 ~ 10.850) | <0.001 | 3.950 (1.700 ~ 9.180) | 0.001 |
| P for trend | 1.520 (1.230 ~ 1.880) | <0.001 | 1.510 (1.220 ~ 1.870) | <0.001 | 1.450 (1.170 ~ 1.800) | <0.001 |
| **Patients with Pre-DM** | |  |  |  |  |  |
| Group 1 | 1.00 (Reference) |  | 1.00 (Reference) |  | 1.00 (Reference) |  |
| Group 2 | 1.890 (0.860 ~ 4.150) | 0.110 | 1.950 (0.880 ~ 4.320) | 0.098 | 1.00 (Reference) | 0.210 |
| Group 3 | 1.410 (0.310 ~ 6.420) | 0.650 | 1.490 (0.330 ~ 6.740) | 0.600 | 1.680 (0.750 ~ 3.780) | 0.720 |
| Group 4 | 2.880 (1.330 ~ 6.240) | 0.007 | 2.910 (1.340 ~ 6.320) | 0.006 | 1.320 (0.290 ~ 6.010) | 0.025 |
| P for trend | 1.350 (1.070 ~ 1.700) | 0.011 | 1.360 (1.080 ~ 1.710) | 0.010 | 2.450 (1.120 ~ 5.360) | 0.034 |
| **Patients with DM** |  |  |  |  |  |  |
| Group 1 | 1.00 (Reference) |  | 1.00 (Reference) |  | 1.00 (Reference) |  |
| Group 2 | 1.420 (0.790 ~ 2.550) | 0.240 | 1.380 (0.770 ~ 2.480) | 0.280 | 1.240 (0.680 ~ 2.260) | 0.480 |
| Group 3 | 2.310 (1.070 ~ 4.000) | 0.032 | 2.220 (1.030 ~ 4.790) | 0.042 | 1.890 (1.021 ~ 4.110) | 0.047 |
| Group 4 | 2.550 (1.450 ~ 4.490) | 0.001 | 2.470 (1.400 ~ 4.360) | 0.002 | 1.980 (1.110 ~ 3.530) | 0.021 |
| P for trend | 1.350 (1.150 ~ 1.580) | <0.001 | 1.340 (1.140 ~ 1.570) | <0.001 | 1.250 (1.060 ~ 1.480) | 0.008 |
| **90-day mortality** |  |  |  |  |  |  |
| **Overall** |  |  |  |  |  |  |
| Group 1 | 1.00 (Reference) |  | 1.00 (Reference) |  | 1.00 (Reference) |  |
| Group 2 | 1.310 (0.910 ~ 1.890) | 0.150 | 1.290 (0.890 ~ 1.870) | 0.180 | 1.340 (0.920 ~ 1.950) | 0.130 |
| Group 3 | 2.380 (1.310 ~ 4.330) | 0.004 | 2.490 (1.370 ~ 4.530) | 0.003 | 2.540 (1.390 ~ 4.640) | 0.002 |
| Group 4 | 2.580 (1.380 ~ 4.820) | 0.003 | 2.790 (1.490 ~ 5.220) | 0.001 | 2.760 (1.470 ~ 5.180) | 0.002 |
| P for trend | 1.230 (1.110 ~ 1.360) | <0.001 | 1.220 (1.100 ~ 1.350) | <0.001 | 1.210 (1.090 ~ 1.340) | <0.001 |
| **Patients with NGR** | | | | | | |
| Group 1 | 1.00 (Reference) |  | 1.00 (Reference) |  | 1.00 (Reference) |  |
| Group 2 | 1.490 (0.730 ~ 3.040) | 0.270 | 1.430 (0.700 ~ 2.920) | 0.320 | 1.550 (0.750 ~ 3.200) | 0.240 |
| Group 3 | 2.290 (0.710 ~ 7.380) | 0.160 | 2.450 (0.760 ~ 7.890) | 0.140 | 2.580 (0.790 ~ 8.420) | 0.120 |
| Group 4 | 2.950 (1.460 ~ 5.960) | 0.003 | 2.770 (1.370 ~ 5.600) | 0.005 | 2.730 (1.340 ~ 5.560) | 0.006 |
| P for trend | 1.410 (1.160 ~ 1.710) | <0.001 | 1.390 (1.140 ~ 1.690) | 0.001 | 1.360 (1.110 ~ 1.660) | 0.003 |
| **Patients with Pre-DM** | | | | | | |
| Group 1 | 1.00 (Reference) |  | 1.00 (Reference) |  | 1.00 (Reference) |  |
| Group 2 | 1.470 (0.700 ~ 3.080) | 0.310 | 1.500 (0.710 ~ 3.160) | 0.290 | 1.510 (0.660 ~ 3.450) | 0.300 |
| Group 3 | 1.280 (0.280 ~ 5.850) | 0.750 | 1.310 (0.290 ~ 5.960) | 0.730 | 1.480 (0.320 ~ 6.890) | 0.630 |
| Group 4 | 2.190 (1.060 ~ 4.520) | 0.034 | 2.220 (1.070 ~ 4.600) | 0.032 | 2.370 (1.050 ~ 5.350) | 0.024 |
| P for trend | 1.270 (1.020 ~ 1.580) | 0.032 | 1.280 (1.030 ~ 1.590) | 0.028 | 1.290 (1.030 ~ 1.610) | 0.026 |
| **Patients with DM** |  |  |  |  |  |  |
| Group 1 | 1.00 (Reference) |  | 1.00 (Reference) |  | 1.00 (Reference) |  |
| Group 2 | 1.180 (0.690 ~ 2.020) | 0.550 | 1.100 (0.640 ~ 1.890) | 0.740 | 1.080 (0.620 ~ 1.880) | 0.790 |
| Group 3 | 2.190 (1.410 ~ 4.100) | 0.008 | 2.170 (1.390 ~ 4.080) | 0.009 | 2.650 (1.170 ~ 4.800) | 0.020 |
| Group 4 | 2.380 (1.650 ~ 4.430) | 0.018 | 2.280 (1.580 ~ 4.290) | 0.015 | 2.750 (1.460 ~ 5.180) | 0.012 |
| P for trend | 1.120 (1.070 ~ 1.290) | 0.006 | 1.130 (1.080 ~ 1.300) | 0.005 | 1.090 (1.040 ~ 1.270) | 0.008 |

Model 1: unadjusted;

Model 2: adjusted for age and sex

Model 3: adjusted for age, sex, HTN, AKI, LC, HB, RBC, WBC, PT, Creatinine and BUN

**Table S8 The association of the combination of PNI and SII with all-cause mortality after excluding individuals with hypoglycemic episodes**

| **Variables** | **Model 1** | | **Model 2** |  | **Model 3** | |
| --- | --- | --- | --- | --- | --- | --- |
|  | **HR (95%CI)** | **P** | **HR (95%CI)** | **P** | **HR (95%CI)** | **P** |
| **28-day mortality** |  |  |  |  |  |  |
| **Overall** |  |  |  |  |  |  |
| Group 1 | 1.00 (Reference) |  | 1.00 (Reference) |  | 1.00 (Reference) |  |
| Group 2 | 1.752 (1.150 ~ 2.669) | 0.009 | 1.684 (1.102 ~ 2.573) | 0.016 | 1.512 (1.008 ~ 2.333) | 0.042 |
| Group 3 | 2.401 (1.280 ~ 4.503) | 0.006 | 2.520 (1.340 ~ 4.740) | 0.004 | 2.340 (1.240 ~ 4.420) | 0.004 |
| Group 4 | 2.980 (1.990 ~ 4.462) | <0.001 | 2.950 (1.960 ~ 4.440) | <0.001 | 2.470 (1.630 ~ 3.740) | <0.001 |
| P for trend | 1.421 (1.275 ~ 1.585) | <0.001 | 1.370 (1.230 ~ 1.530) | <0.001 | 1.290 (1.150 ~ 1.450) | <0.001 |
| **Patients with NGR** | |  |  |  |  |  |
| Group 1 | 1.00 (Reference) |  | 1.00 (Reference) |  | 1.00 (Reference) |  |
| Group 2 | 2.450 (1.060 ~ 5.660) | 0.036 | 2.360 (1.030 ~ 5.460) | 0.042 | 2.110 (1.010 ~ 4.900) | 0.047 |
| Group 3 | 2.520 (0.740 ~ 8.590) | 0.140 | 2.650 (0.780 ~ 9.010) | 0.120 | 2.780 (0.800 ~ 9.660) | 0.110 |
| Group 4 | 4.860 (2.140 ~ 11.040) | <0.001 | 4.720 (2.070 ~ 10.760) | <0.001 | 4.020 (1.750 ~ 9.240) | 0.001 |
| P for trend | 1.520 (1.240 ~ 1.860) | <0.001 | 1.510 (1.230 ~ 1.850) | <0.001 | 1.450 (1.180 ~ 1.780) | <0.001 |
| **Patients with Pre-DM** | |  |  |  |  |  |
| Group 1 | 1.00 (Reference) |  | 1.00 (Reference) |  | 1.00 (Reference) |  |
| Group 2 | 1.860 (0.850 ~ 4.070) | 0.120 | 1.970 (0.900 ~ 4.310) | 0.090 | 1.620 (0.670 ~ 3.920) | 0.280 |
| Group 3 | 1.380 (0.300 ~ 6.350) | 0.680 | 1.490 (0.320 ~ 6.890) | 0.610 | 1.290 (0.270 ~ 6.170) | 0.750 |
| Group 4 | 2.850 (1.320 ~ 6.150) | 0.008 | 2.890 (1.340 ~ 6.240) | 0.007 | 2.420 (1.030 ~ 5.680) | 0.042 |
| P for trend | 1.350 (1.070 ~ 1.700) | 0.011 | 1.350 (1.070 ~ 1.710) | 0.011 | 1.280 (1.010 ~ 1.630) | 0.043 |
| **Patients with DM** |  |  |  |  |  |  |
| Group 1 | 1.00 (Reference) |  | 1.00 (Reference) |  | 1.00 (Reference) |  |
| Group 2 | 1.420 (0.790 ~ 2.550) | 0.240 | 1.370 (0.760 ~ 2.470) | 0.290 | 1.220 (0.660 ~ 2.260) | 0.520 |
| Group 3 | 2.280 (1.050 ~ 4.950) | 0.037 | 2.180 (1.034 ~ 4.750) | 0.041 | 1.840 (0.840 ~ 4.030) | 0.043 |
| Group 4 | 2.510 (1.430 ~ 4.410) | 0.001 | 2.420 (1.380 ~ 4.250) | 0.002 | 1.890 (1.030 ~ 3.470) | 0.040 |
| P for trend | 1.340 (1.150 ~ 1.560) | <0.001 | 1.330 (1.140 ~ 1.550) | <0.001 | 1.250 (1.060 ~ 1.470) | 0.008 |
| **90-day mortality** |  |  |  |  |  |  |
| **Overall** |  |  |  |  |  |  |
| Group 1 | 1.00 (Reference) |  | 1.00 (Reference) |  | 1.00 (Reference) |  |
| Group 2 | 1.420 (0.980 ~ 2.060) | 0.064 | 1.380 (0.950 ~ 2.000) | 0.088 | 1.450 (0.990 ~ 2.120) | 0.055 |
| Group 3 | 2.310 (1.270 ~ 4.200) | 0.006 | 2.450 (1.350 ~ 4.450) | 0.003 | 2.520 (1.380 ~ 4.600) | 0.003 |
| Group 4 | 2.580 (1.380 ~ 4.820) | <0.001 | 2.820 (1.510 ~ 5.270) | <0.001 | 2.780 (1.480 ~ 5.220) | <0.001 |
| P for trend | 1.220 (1.100 ~ 1.350) | <0.001 | 1.210 (1.090 ~ 1.340) | <0.001 | 1.190 (1.070 ~ 1.330) | 0.001 |
| **Patients with NGR** | | | | | | |
| Group 1 | 1.00 (Reference) |  | 1.00 (Reference) |  | 1.00 (Reference) |  |
| Group 2 | 1.620 (0.800 ~ 3.280) | 0.180 | 1.550 (0.760 ~ 3.160) | 0.230 | 1.720 (0.840 ~ 3.520) | 0.140 |
| Group 3 | 2.280 (0.710 ~ 7.320) | 0.160 | 2.450 (0.760 ~ 7.890) | 0.130 | 2.590 (0.800 ~ 8.380) | 0.110 |
| Group 4 | 3.010 (1.490 ~ 6.080) | 0.002 | 2.820 (1.390 ~ 5.720) | 0.004 | 2.770 (1.360 ~ 5.640 | 0.005 |
| P for trend | 1.410 (1.160 ~ 1.710) | <0.001 | 1.390 (1.140 ~ 1.690) | <0.001 | 1.360 (1.110 ~ 1.660) | 0.003 |
| **Patients with Pre-DM** | | | | | | |
| Group 1 | 1.00 (Reference) |  | 1.00 (Reference) |  | 1.00 (Reference) |  |
| Group 2 | 1.490 (0.720 ~ 3.080) | 0.280 | 1.520 (0.730 ~ 3.160) | 0.260 | 1.510 (0.660 ~ 3.450) | 0.330 |
| Group 3 | 1.290 (0.280 ~ 5.890) | 0.750 | 1.320 (0.290 ~ 6.010) | 0.730 | 1.480 (0.320 ~ 6.890) | 0.620 |
| Group 4 | 2.210 (1.070 ~ 4.560) | 0.032 | 2.250 (1.090 ~ 4.650) | 0.029 | 2.370 (1.050 ~ 5.350) | 0.038 |
| P for trend | 1.260 (1.020 ~ 1.560) | 0.033 | 1.270 (1.030 ~ 1.570) | 0.027 | 1.290 (1.030 ~ 1.610) | 0.025 |
| **Patients with DM** |  |  |  |  |  |  |
| Group 1 | 1.00 (Reference) |  | 1.00 (Reference) |  | 1.00 (Reference) |  |
| Group 2 | 1.180 (0.690 ~ 2.020) | 0.550 | 1.100 (0.640 ~ 1.890) | 0.730 | 1.080 (0.610 ~ 1.910) | 0.790 |
| Group 3 | 2.150 (1.390 ~ 4.080) | 0.009 | 2.140 (1.370 ~ 4.050) | 0.008 | 2.660 (1.160 ~ 4.890) | 0.020 |
| Group 4 | 2.350 (1.630 ~ 4.580) | 0.017 | 2.250 (1.570 ~ 4.480) | 0.015 | 2.760 (1.470 ~ 4.190) | 0.012 |
| P for trend | 1.120 (1.070 ~ 1.290) | 0.008 | 1.130 (1.080 ~ 1.300) | 0.007 | 1.090 (1.050 ~ 1.270) | 0.010 |

Model 1: unadjusted;

Model 2: adjusted for age and sex

Model 3: adjusted for Model 2 plus GCS, CCI, APACHE II score, SpO2, Lactate, PH, Creatinine, BUN, PT, HB, AKI, HF, Hypoglycemic drugs, Mechanical ventilation

Group 1: Low SHR and Low GV (SHR < 1.15 and GV < 24.46); Group 2: Low SHR and High GV (SHR < 1.15 and GV > 24.46); Group 3: High SHR and Low GV (SHR > 1.15 and GV < 24.46); Group 4: High SHR and High GV (SHR > 1.15 and GV > 24.46);

**Table S9 The performance comparison of each ML model in predicting 28-day mortality**

|  | **AUC** | **Sensitivity** | **Specificity** | **Accuracy** | **F1** |
| --- | --- | --- | --- | --- | --- |
| **NGR** |  |  |  |  |  |
| RF | 0.760 | 0.703 | 0.676 | 0.714 | 0.727 |
| XGBoost | 0.853 | 0.754 | 0.730 | 0.772 | 0.783 |
| LGBM | 0.841 | 0.756 | 0.730 | 0.777 | 0.788 |
| CatBoost | 0.850 | 0.761 | 0.748 | 0.768 | 0.774 |
| LR | 0.719 | 0.699 | 0.721 | 0.679 | 0.667 |
| **Pre-DM** |  |  |  |  |  |
| RF | 0.752 | 0.667 | 0.676 | 0.656 | 0.652 |
| XGBoost | 0.894 | 0.916 | 0.928 | 0.848 | 0.837 |
| LGBM | 0.881 | 0.910 | 0.919 | 0.862 | 0.855 |
| CatBoost | 0.848 | 0.798 | 0.793 | 0.799 | 0.802 |
| LR | 0.683 | 0.689 | 0.829 | 0.598 | 0.483 |
| **DM** |  |  |  |  |  |
| RF | 0.672 | 0.591 | 0.588 | 0.623 | 0.624 |
| XGBoost | 0.823 | 0.744 | 0.741 | 0.787 | 0.789 |
| LGBM | 0.868 | 0.727 | 0.710 | 0.779 | 0.786 |
| CatBoost | 0.831 | 0.719 | 0.725 | 0.751 | 0.748 |
| LR | 0.632 | 0.542 | 0.664 | 0.558 | 0.486 |

RF, random forest; XGBoost, extreme gradient boosting; LGBM, light gradient boosting machine; LR, logistic regression,

**Table S10 The association of the PNI and SII with 28-day and 90-day mortality (external cohorts)**

| **Variables** | **Model 1** | | **Model 2** |  | **Model 3** | |
| --- | --- | --- | --- | --- | --- | --- |
|  | **HR (95%CI)** | **P** | **HR (95%CI)** | **P** | **HR (95%CI)** | **P** |
| **28-day mortality** |  |  |  |  |  |  |
| **Overall** |  |  |  |  |  |  |
| PNI |  |  |  |  |  |  |
| T1 | 1.00 (Reference) |  | 1.00 (Reference) |  | 1.00 (Reference) |  |
| T2 | 0.738 (0.543 ~ 1.004) | 0.053 | 0.742 (0.546 ~ 1.010) | 0.058 | 0.782 (0.571 ~ 1.072) | 0.127 |
| T3 | 0.665 (0.470 ~ 0.942) | 0.022 | 0.690 (0.487 ~ 0.978) | 0.037 | 0.678 (0.466 ~ 0.986) | 0.042 |
| P for trend | 0.798 (0.675 ~ 0.944) | 0.008 | 0.817 (0.687 ~ 0.970) | 0.021 | 0.817 (0.680 ~ 0.981) | 0.030 |
| SII |  |  |  |  |  |  |
| T1 | 1.00 (Reference) |  | 1.00 (Reference) |  | 1.00 (Reference) |  |
| T2 | 1.013 (0.700 ~ 1.467) | 0.944 | 1.034 (0.714 ~ 1.498) | 0.859 | 1.107 (0.758 ~ 1.616) | 0.600 |
| T3 | 1.905 (1.368 ~ 2.652) | <0.001 | 1.909 (1.371 ~ 2.659) | <0.001 | 2.006 (1.426 ~ 2.824) | <0.001 |
| P for trend | 1.457 (1.233 ~ 1.721) | <0.001 | 1.432 (1.208 ~ 1.698) | <0.001 | 1.462 (1.229~ 1.739) | <0.001 |
| **Patients with NGR** | |  |  |  |  |  |
| PNI |  |  |  |  |  |  |
| T1 | 1.00 (Reference) |  | 1.00 (Reference) |  | 1.00 (Reference) |  |
| T2 | 0.581 (0.331 ~ 1.019) | 0.058 | 0.569 (0.324 ~ 1.001) | 0.050 | 0.584 (0.327 ~ 1.044) | 0.070 |
| T3 | 0.445 (0.225 ~ 0.883) | 0.020 | 0.464 (0.234 ~ 0.923) | 0.029 | 0.436 (0.213 ~ 0.892) | 0.023 |
| P for trend | 0.649 (0.465 ~ 0.906) | 0.011 | 0.657 (0.469 ~ 0.920) | 0.014 | 0.645 (0.455~ 0.914) | 0.014 |
| SII |  |  |  |  |  |  |
| T1 | 1.00 (Reference) |  | a |  | 1.00 (Reference) |  |
| T2 | 0.747 (0.371 ~ 1.502) | 0.413 | 0.767 (0.381 ~ 1.545) | 0.458 | 0.746 (0.363 ~ 1.536) | 0.427 |
| T3 | 2.024 (1.134 ~ 3.612) | 0.017 | 2.019 (1.131 ~ 3.606) | 0.018 | 1.913 (1.023 ~ 3.576) | 0.042 |
| P for trend | 1.508 (1.101 ~ 2.067) | 0.011 | 1.502 (1.098 ~ 2.055) | 0.011 | 1.465 (1.050 ~ 2.045) | 0.025 |
| **Patients with Pre-DM** | |  |  |  |  |  |
| PNI |  |  |  |  |  |  |
| T1 | 1.00 (Reference) |  | 1.00 (Reference) |  | 1.00 (Reference) |  |
| T2 | 0.735 (0.372 ~ 1.454) | 0.377 | 0.748 (0.375 ~ 1.492) | 0.410 | 0.801 (0.392 ~ 1.639) | 0.544 |
| T3 | 0.574 (0.276 ~ 1.194) | 0.137 | 0.556 (0.267 ~ 1.158) | 0.117 | 0.575 (0.254 ~ 1.301) | 0.184 |
| P for trend | 0.756 (0.525 ~ 1.089) | 0.133 | 0.746 (0.518 ~ 1.073) | 0.115 | 0.762 (0.511 ~ 1.137) | 0.183 |
| SII |  |  |  |  |  |  |
| T1 | 1.00 (Reference) |  | / |  | 1.00 (Reference) |  |
| T2 | 1.040 (0.489 ~ 2.212) | 0.920 | 1.085 (0.509 ~ 2.313) | 0.833 | 1.069 (0.483 ~ 2.366) | 0.870 |
| T3 | 1.649 (0.814 ~ 3.342) | 0.165 | 1.735 (0.852 ~ 3.532) | 0.129 | 1.812 (0.856 ~ 3.837) | 0.120 |
| P for trend | 1.302 (0.905 ~ 1.873) | 0.155 | 1.334 (0.926 ~ 1.921) | 0.122 | 1.375 (0.937 ~ 2.019) | 0.104 |
| **Patients with DM** |  |  |  |  |  |  |
| PNI |  |  |  |  |  |  |
| T1 | 1.00 (Reference) |  | 1.00 (Reference) |  | 1.00 (Reference) |  |
| T2 | 0.852 (0.549 ~ 1.323) | 0.476 | 0.904 (0.581 ~ 1.407) | 0.656 | 0.980 (0.620 ~ 1.550) | 0.932 |
| T3 | 0.893 (0.546 ~ 1.459) | 0.651 | 0.990 (0.603 ~ 1.627) | 0.969 | 0.998 (0.586 ~ 1.698) | 0.994 |
| P for trend | 0.930 (0.731 ~ 1.184) | 0.555 | 0.981 (0.769 ~ 1.251) | 0.876 | 0.996 (0.769 ~ 1.291) | 0.977 |
| SII |  |  |  |  |  |  |
| T1 | 1.00 (Reference) |  | 1.00 (Reference) |  | 1.00 (Reference) |  |
| T2 | 1.189 (0.687 ~ 2.055) | 0.536 | 1.226 (0.708 ~ 2.121) | 0.467 | 1.359 (0.774 ~ 2.386) | 0.285 |
| T3 | 1.940 (1.176 ~ 3.202) | 0.009 | 1.950 (1.179 ~ 3.225) | 0.009 | 2.089 (1.235 ~ 3.532) | 0.006 |
| P for trend | 1.432 (1.118 ~ 1.834) | 0.004 | 1.428 (1.115 ~ 1.829) | 0.005 | 1.461 (1.132 ~ 1.885) | 0.004 |
| **90-day mortality** |  |  |  |  |  |  |
| **Overall** |  |  |  |  |  |  |
| PNI |  |  |  |  |  |  |
| T1 | 1.00 (Reference) |  | 1.00 (Reference) |  | 1.00 (Reference) |  |
| T2 | 0.776 (0.586 ~ 1.026) | 0.075 | 0.786 (0.594 ~ 1.040) | 0.092 | 0.809 (0.608 ~ 1.078) | 0.148 |
| T3 | 0.668 (0.483 ~ 0.924) | 0.015 | 0.699 (0.505 ~ 0.967) | 0.031 | 0.682 (0.483 ~ 0.962) | 0.029 |
| P for trend | 0.811 (0.692 ~ 0.949) | 0.009 | 0.828 (0.707 ~ 0.970) | 0.019 | 0.823 (0.697 ~ 0.972) | 0.022 |
| SII |  |  |  |  |  |  |
| T1 | 1.00 (Reference) |  | 1.00 (Reference) |  | 1.00 (Reference) |  |
| T2 | 1.178 (0.847 ~ 1.640) | 0.330 | 1.224 (0.879 ~ 1.704) | 0.232 | 1.271 (0.905 ~ 1.783) | 0.166 |
| T3 | 1.790 (1.318 ~ 2.430) | <0.001 | 1.808 (1.331 ~ 2.457) | <0.001 | 1.835 (1.339 ~ 2.514) | <0.001 |
| P for trend | 1.358 (1.165 ~ 1.583) | <0.001 | 1.359 (1.167 ~ 1.584) | <0.001 | 1.365 (1.168 ~ 1.595) | <0.001 |
| **Patients with NGR** | | | | | | |
| PNI |  |  |  |  |  |  |
| T1 | 1.00 (Reference) |  | 1.00 (Reference) |  | 1.00 (Reference) |  |
| T2 | 0.689 (0.422 ~ 1.125) | 0.137 | 0.683 (0.417 ~ 1.117) | 0.128 | 0.691 (0.417 ~ 1.146) | 0.152 |
| T3 | 0.519 (0.282 ~ 0.955) | 0.035 | 0.562 (0.304 ~ 0.938) | 0.032 | 0.519 (0.273 ~ 0.987) | 0.046 |
| P for trend | 0.714 (0.533 ~ 0.957) | 0.024 | 0.735 (0.547 ~ 0.989) | 0.042 | 0.715 (0.525 ~ 0.973) | 0.033 |
| SII |  |  |  |  |  |  |
| T1 | 1.00 (Reference) |  | 1.00 (Reference) |  | 1.00 (Reference) |  |
| T2 | 0.888 (0.493 ~ 1.600) | 0.694 | 0.937 (0.519 ~ 1.691) | 0.829 | 0.922 (0.502 ~ 1.693) | 0.794 |
| T3 | 1.908 (1.138 ~ 3.198) | 0.014 | 1.901 (1.134 ~ 3.188) | 0.015 | 1.804 (1.042 ~ 3.124) | 0.035 |
| P for trend | 1.426 (1.084 ~ 1.876) | 0.011 | 1.417 (1.080 ~ 1.860) | 0.012 | 1.382 (1.039 ~ 1.839) | 0.026 |
| **Patients with Pre-DM** | | | | | | |
| PNI |  |  |  |  |  |  |
| T1 | 1.00 (Reference) |  | 1.00 (Reference) |  | 1.00 (Reference) |  |
| T2 | 0.731 (0.390 ~ 1.371) | 0.329 | 0.758 (0.402 ~ 1.430) | 0.392 | 0.788 (0.406 ~ 1.531) | 0.483 |
| T3 | 0.492 (0.241 ~ 1.002) | 0.051 | 0.483 (0.237 ~ 0.984) | 0.045 | 0.479 (0.216 ~ 1.061) | 0.069 |
| P for trend | 0.704 (0.497 ~ 0.998) | 0.048 | 0.701 (0.496 ~ 0.991) | 0.044 | 0.703 (0.480 ~ 1.030) | 0.071 |
| SII |  |  |  |  |  |  |
| T1 | 1.00 (Reference) |  | 1.00 (Reference) |  | 1.00 (Reference) |  |
| T2 | 1.367 (0.669 ~ 2.794) | 0.392 | 1.443 (0.703 ~ 2.960) | 0.317 | 1.472 (0.689 ~ 3.145) | 0.318 |
| T3 | 1.854 (0.927 ~ 3.706) | 0.081 | 1.968 (0.979 ~ 3.959) | 0.057 | 2.134 (1.015 ~ 4.487) | 0.045 |
| P for trend | 1.361 (0.966 ~ 1.917) | 0.078 | 1.400 (0.992 ~ 1.974) | 0.055 | 1.460 (1.014 ~ 2.102) | 0.042 |
| **Patients with DM** | | | | | | |
| PNI |  |  |  |  |  |  |
| T1 | 1.00 (Reference) |  | 1.00 (Reference) |  | 1.00 (Reference) |  |
| T2 | 0.837 (0.553 ~ 1.266) | 0.400 | 0.891 (0.588 ~ 1.350) | 0.588 | 0.970 (0.630 ~ 1.493) | 0.890 |
| T3 | 0.893 (0.565 ~ 1.410) | 0.627 | 0.979 (0.618 ~ 1.549) | 0.927 | 0.980 (0.604 ~ 1.590) | 0.934 |
| P for trend | 0.927 (0.741 ~ 1.161) | 0.510 | 0.974 (0.778 ~ 1.219) | 0.816 | 0.987 (0.780~ 1.250) | 0.914 |
| SII |  |  |  |  |  |  |
| T1 | 1.00 (Reference) |  | 1.00 (Reference) |  | 1.00 (Reference) |  |
| T2 | 1.321 (0.806 ~ 2.164) | 0.269 | 1.402 (0.853 ~ 2.302) | 0.182 | 1.547 (0.929 ~ 2.576) | 0.094 |
| T3 | 1.724 (1.084 ~ 2.743) | 0.021 | 1.761 (1.104 ~ 2.810) | 0.018 | 1.860 (1.145 ~ 3.021) | 0.012 |
| P for trend | 1.312 (1.049 ~ 1.641) | 0.017 | 1.316 (1.053 ~ 1.645) | 0.016 | 1.336 (1.063 ~ 1.680) | 0.013 |

Model 1: unadjusted;

Model 2: adjusted for age and sex

Model 3: adjusted for age, sex, HTN, AKI, LC, HB, RBC, WBC, PT, Creatinine and BUN

**Table S11 The association of the combination of PNI and SII with all-cause mortality (external cohorts)**

| **Variables** | **Model 1** | | **Model 2** |  | **Model 3** | |
| --- | --- | --- | --- | --- | --- | --- |
|  | **HR (95%CI)** | **P** | **HR (95%CI)** | **P** | **HR (95%CI)** | **P** |
| **28-day mortality** |  |  |  |  |  |  |
| **Overall** |  |  |  |  |  |  |
| Group 1 | 1.00 (Reference) |  | 1.00 (Reference) |  | 1.00 (Reference) |  |
| Group 2 | 1.288 (0.851 ~ 1.949) | 0.231 | 1.251 (0.826 ~ 1.894) | 0.291 | 1.293 (0.835 ~ 2.002) | 0.250 |
| Group 3 | 2.743 (1.459 ~ 5.156) | 0.002 | 2.830 (1.505 ~ 5.323) | 0.001 | 3.008 (1.588 ~ 5.698) | 0.001 |
| Group 4 | 2.200 (1.465 ~ 3.304) | <0.001 | 2.123 (1.413 ~ 3.191) | <0.001 | 2.185 (1.429 ~ 3.341) | <0.001 |
| P for trend | 1.473 (1.187 ~ 1.828) | <0.001 | 1.299 (1.159 ~ 1.457) | <0.001 | 1.433 (1.143 ~ 1.798) | 0.002 |
| **Patients with NGR** | |  |  |  |  |  |
| Group 1 | 1.00 (Reference) |  | 1.00 (Reference) |  | 1.00 (Reference) |  |
| Group 2 | 1.717 (0.741 ~ 3.982) | 0.208 | 1.651 (0.711 ~ 3.835) | 0.244 | 1.733 (0.733 ~ 4.101) | 0.211 |
| Group 3 | 3.170 (0.927 ~ 10.844) | 0.066 | 3.330 (0.973 ~ 11.397) | 0.055 | 3.344 (0.964 ~ 11.602) | 0.057 |
| Group 4 | 3.485 (1.522~ 7.983) | 0.003 | 3.297 (1.437 ~ 7.563) | 0.005 | 3.244 (1.391~ 7.566) | 0.006 |
| P for trend | 1.473 (1.187~ 1.828) | <0.001 | 1.454 (1.171~ 1.805) | 0.001 | 1.433 (1.143~ 1.798) | 0.002 |
| **Patients with Pre-DM** | |  |  |  |  |  |
| Group 1 | 1.00 (Reference) |  | 1.00 (Reference) |  | 1.00 (Reference) |  |
| Group 2 | 1.299 (0.592 ~ 2.850) | 0.514 | 1.365 (0.621 ~ 3.000) | 0.438 | 1.347 (0.571 ~ 3.175) | 0.497 |
| Group 3 | 1.380 (0.302 ~ 6.301) | 0.678 | 1.482 (0.323 ~ 6.795) | 0.612 | 1.692 (0.359 ~ 7.967) | 0.506 |
| Group 4 | 1.979 (0.903 ~ 4.339) | 0.088 | 2.089 (0.951 ~ 4.589) | 0.067 | 2.174 (0.923 ~ 5.122) | 0.076 |
| P for trend | 1.247 (0.978 ~ 1.589) | 0.075 | 1.265 (0.992 ~ 1.612) | 0.058 | 1.287 (0.997 ~ 1.662) | 0.053 |
| **Patients with DM** | | | | | | |
| Group 1 | 1.00 (Reference) |  | 1.00 (Reference) |  | 1.00 (Reference) |  |
| Group 2 | 1.139 (0.619 ~ 2.095) | 0.676 | 1.047 (0.567 ~ 1.932) | 0.884 | 1.090 (0.569 ~ 2.088) | 0.796 |
| Group 3 | 3.448 (1.446 ~ 8.226) | 0.005 | 3.485 (1.455 ~ 8.349) | 0.005 | 3.373 (1.375 ~ 8.270) | 0.008 |
| Group 4 | 1.796 (0.990 ~ 3.257) | 0.054 | 1.660 (0.912 ~ 3.019) | 0.097 | 1.705 (0.909 ~ 3.199) | 0.096 |
| P for trend | 1.242 (1.055 ~ 1.461) | 0.009 | 1.227 (1.041 ~ 1.446) | 0.015 | 1.234 (1.040 ~ 1.463) | 0.016 |
| **90-day mortality** |  |  |  |  |  |  |
| **Overall** |  |  |  |  |  |  |
| Group 1 | 1.00 (Reference) |  | 1.00 (Reference) |  | 1.00 (Reference) |  |
| Group 2 | 1.403 (0.961 ~ 2.048) | 0.080 | 1.357 (0.929 ~ 1.982) | 0.114 | 1.417 (0.953 ~ 2.107) | 0.085 |
| Group 3 | 2.661 (1.456 ~ 4.861) | 0.001 | 2.763 (1.511 ~ 5.051) | 0.001 | 2.889 (1.570 ~ 5.315) | 0.001 |
| Group 4 | 2.024 (1.385 ~ 2.956) | <0.001 | 1.945 (1.331 ~ 2.842) | 0.001 | 1.972 (1.333 ~ 2.918) | 0.001 |
| P for trend | 1.242 (1.119 ~ 1.379) | <0.001 | 1.232 (1.110 ~ 1.368) | <0.001 | 1.230 (1.106 ~ 1.368) | <0.001 |
| **Patients with NGR** | | | | | | |
| Group 1 | 1.00 (Reference) |  | 1.00 (Reference) |  | 1.00 (Reference) |  |
| Group 2 | 1.510 (0.745 ~ 3.061) | 0.253 | 1.417 (0.697 ~ 2.881) | 0.335 | 1.521 (0.734 ~ 3.148) | 0.259 |
| Group 3 | 2.357 (0.738 ~ 7.532) | 0.148 | 2.542 (0.795 ~ 8.128) | 0.116 | 2.527 (0.780 ~ 8.191) | 0.122 |
| Group 4 | 2.785 (1.372 ~ 5.655) | 0.005 | 2.540 (1.247 ~ 5.174) | 0.010 | 2.530 (1.226 ~ 5.218) | 0.012 |
| P for trend | 1.385 (1.143~ 1.678) | 0.001 | 1.356 (1.118 ~ 1.643) | 0.002 | 1.334 (1.094 ~ 1.627) | 0.004 |
| **Patients with Pre-DM** | | | | | | |
| Group 1 | 1.00 (Reference) |  | 1.00 (Reference) |  | 1.00 (Reference) |  |
| Group 2 | 1.573 (0.737 ~ 3.356) | 0.242 | 1.634 (0.764 ~ 3.495) | 0.205 | 1.638 (0.714 ~ 3.757) | 0.244 |
| Group 3 | 1.360 (0.298 ~ 6.218) | 0.691 | 1.415 (0.309 ~ 6.476) | 0.655 | 1.614 (0.345 ~ 7.558) | 0.543 |
| Group 4 | 2.203 (1.020~ 4.758) | 0.044 | 2.326 (1.074 ~ 5.038) | 0.032 | 2.449 (1.066 ~ 5.565) | 0.035 |
| P for trend | 1.258 (1.001 ~ 1.582) | 0.049 | 1.278 (1.015 ~ 1.607) | 0.037 | 1.299 (1.020 ~ 1.653) | 0.034 |
| **Patients with DM** | | | | | | |
| Group 1 | 1.00 (Reference) |  | 1.00 (Reference) |  | 1.00 (Reference) |  |
| Group 2 | 1.279 (0.730 ~ 2.242) | 0.390 | 1.195 (0.681 ~ 2.099) | 0.534 | 1.238 (0.683 ~ 2.241) | 0.482 |
| Group 3 | 3.668 (1.619 ~ 8.314) | 0.002 | 3.740 (1.643 ~ 8.515) | 0.002 | 3.288 (1.411 ~ 7.663) | 0.006 |
| Group 4 | 1.608 (0.690~ 4.733) | 0.014 | 1.506 (0.859~ 2.639) | 0.153 | 1.529 (0.854 ~ 2.738) | 0.153 |
| P for trend | 1.158 (0.919 ~ 2.814) | 0.096 | 1.144 (0.984 ~ 1.330) | 0.080 | 1.145 (0.987 ~ 1.337) | 0.082 |

Molde 1: unadjusted;

Model 2: adjusted for age and sex

Model 3: adjusted for age, sex, HTN, AKI, LC, HB, RBC, WBC, PT, Creatinine and BUN


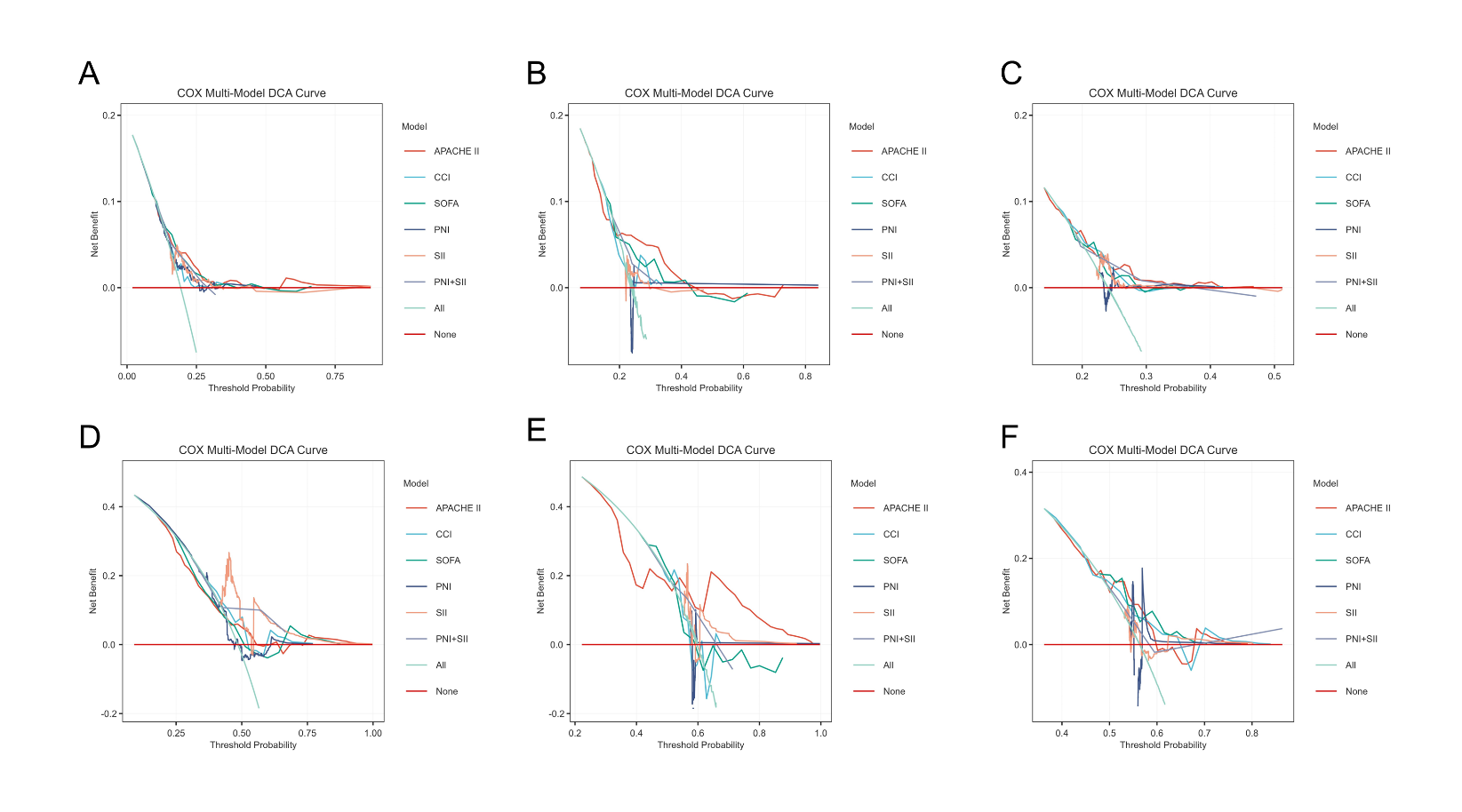


**Figure S1** Decision Curve Analysis for evaluating the net clinical benefit of different predictive models. **A.** Decision curve analysis comparing the net benefit of the PNI, SII, PNI+SII combined model versus APACHE II, CCI, and SOFA scores for predicting 28-day mortality in NGR patients. **B.** Decision curve analysis comparing the net benefit of the PNI, SII, PNI+SII combined model versus APACHE II, CCI, and SOFA scores for predicting 28-day mortality in Pre-DM patients. **C.** Decision curve analysis comparing the net benefit of the PNI, SII, PNI+SII combined model versus APACHE II, CCI, and SOFA scores for predicting 28-day mortality in DM patients. **D.** Decision curve analysis comparing the net benefit of the PNI, SII, PNI+SII combined model versus APACHE II, CCI, and SOFA scores for predicting 90-day mortality in NGR patients. **E.** Decision curve analysis comparing the net benefit of the PNI, SII, PNI+SII combined model versus APACHE II, CCI, and SOFA scores for predicting 90-day mortality in Pre-DM patients. **F.** Decision curve analysis comparing the net benefit of the PNI, SII, PNI+SII combined model versus APACHE II, CCI, and GCS scores for predicting 90-day mortality in DM patients.

**
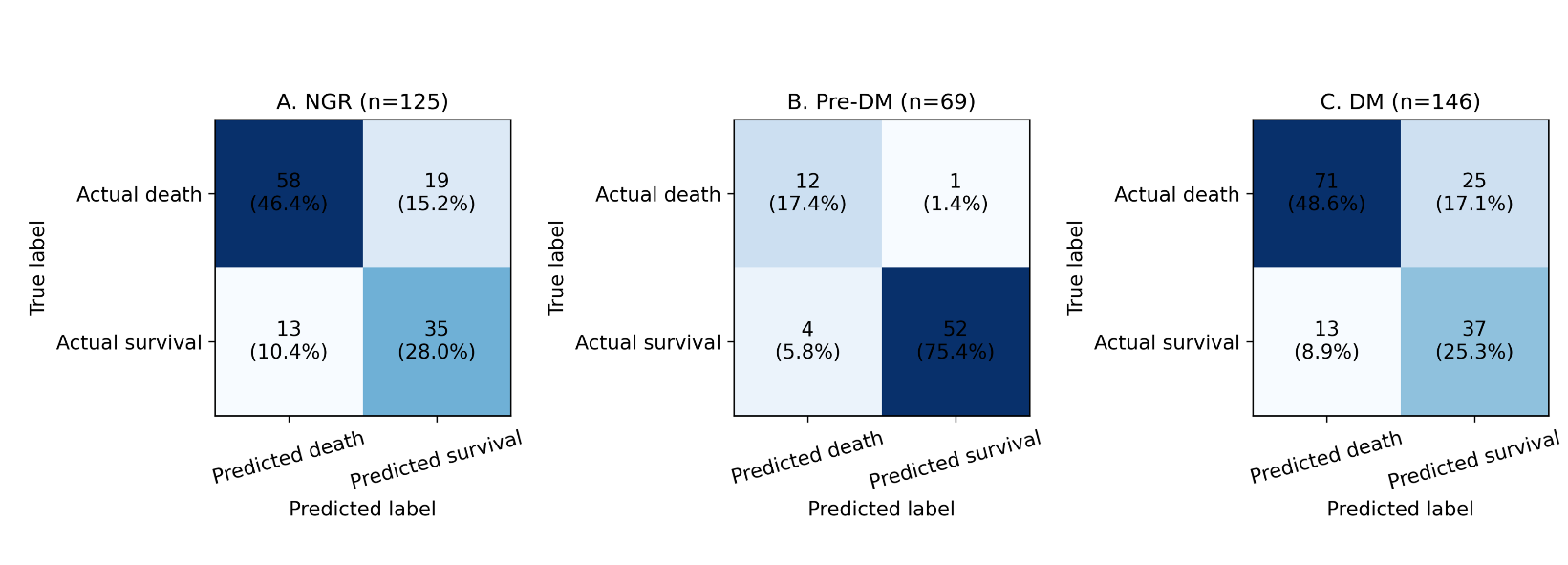
Figure S2** Confusion matrices of the XGBoost model in the internal test cohort across glycemic subgroups. Confusion matrices (A–C) illustrate the distribution of true positives, false positives, false negatives, and true negatives at a fixed classification threshold. Values represent absolute counts, with percentages shown in parentheses.


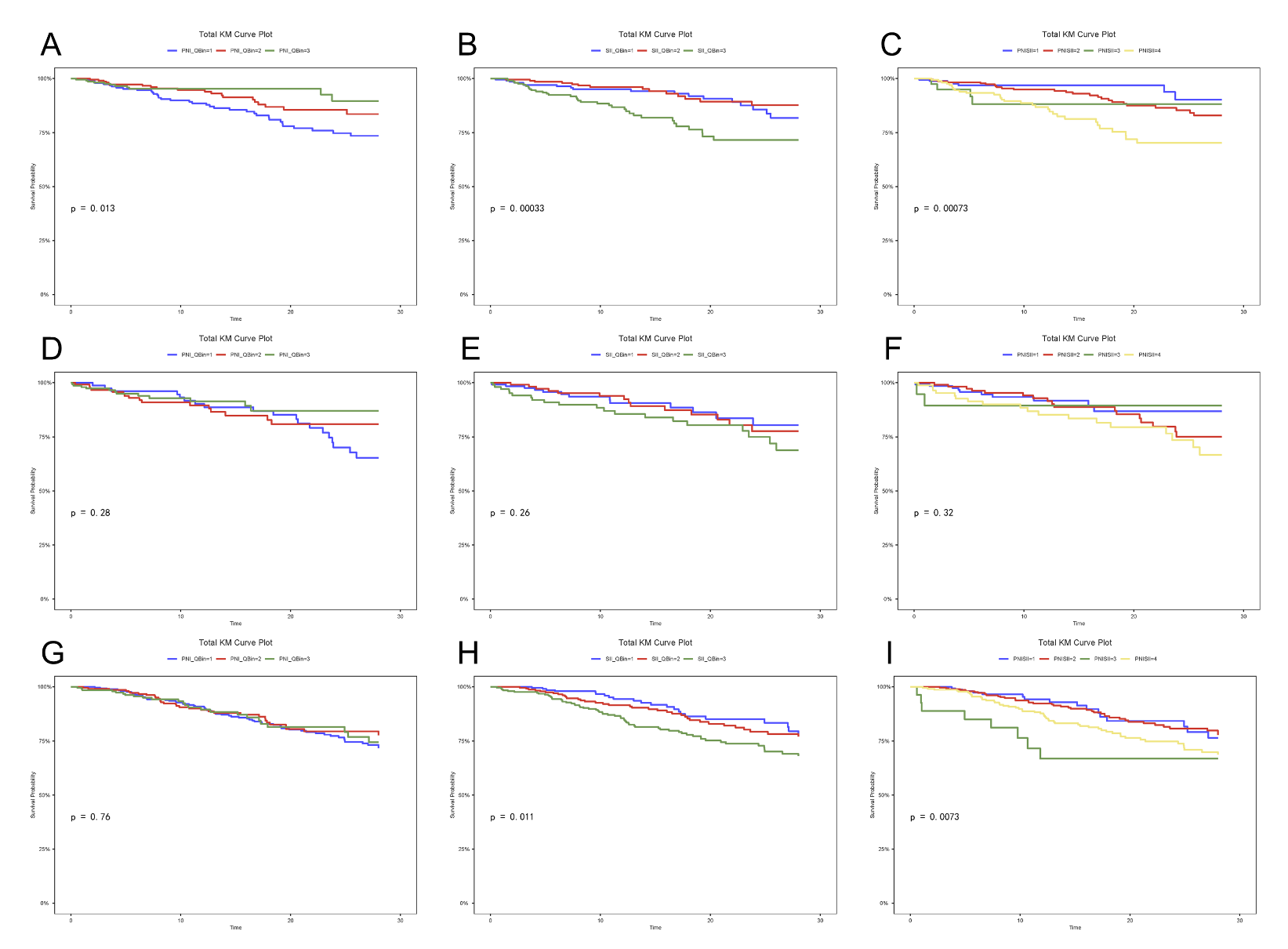


**Figure S3** Kaplan-Meier curves of PNI, SII, and their combination for 28-day mortality (external cohorts). (**A**–**C**) patients with NGR; (**D**–**F**) patients with Pre-DM; (**G**–**I**) patients with DM


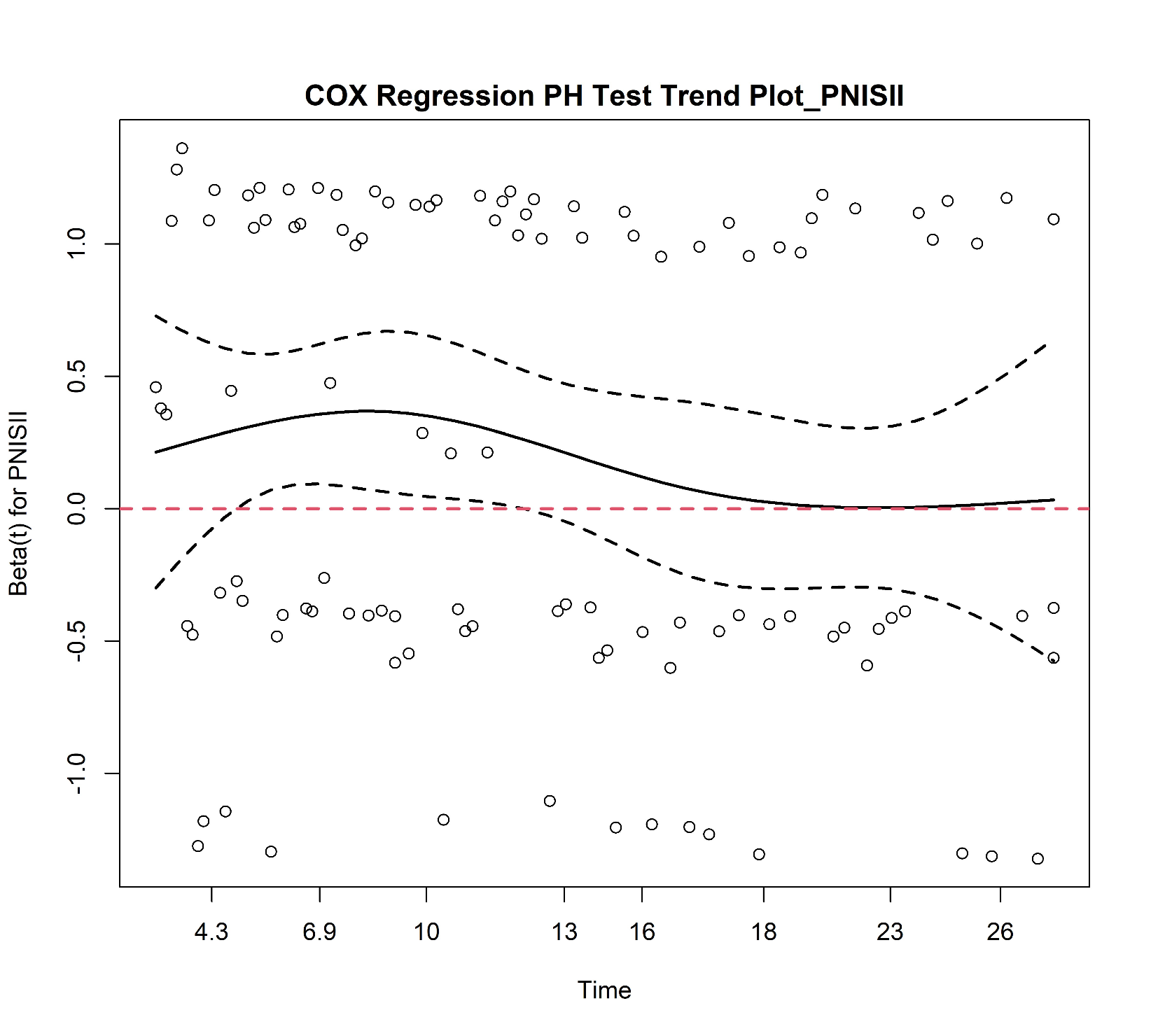
**Figure S4** The trends of the proportional - hazards assumption test based on the COX regression model (external cohorts).


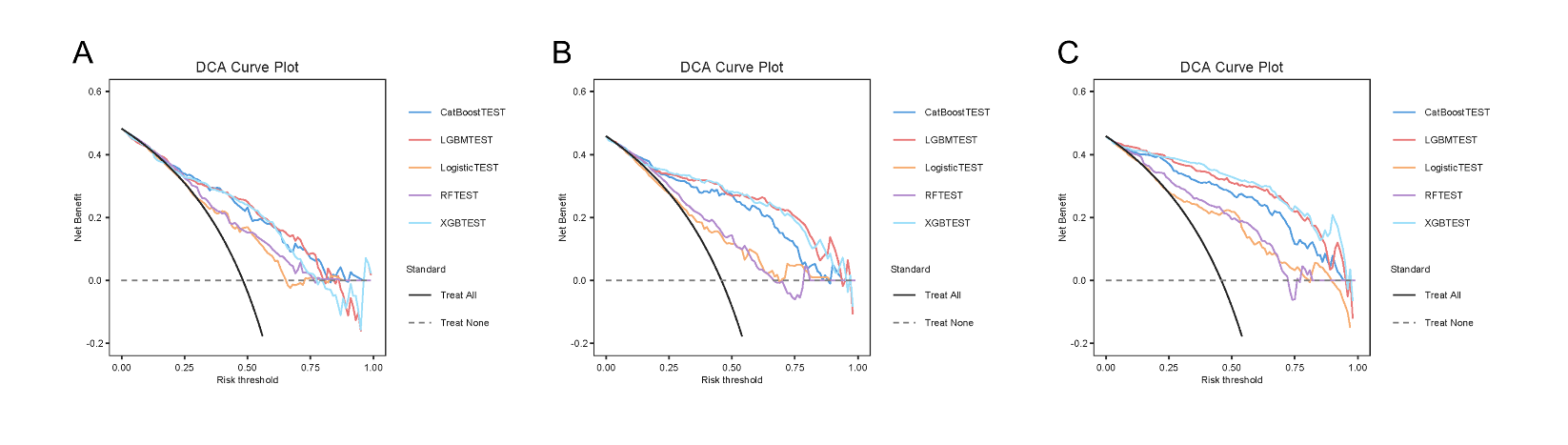


**Figure S5** Decision Curve Analysis for Evaluating the Clinical Utility of the PNI+SII Combined Model Across Different Risk Subgroups. **A.** patients with NGR; **B.** patients with Pre-DM; **C.** patients with DM.


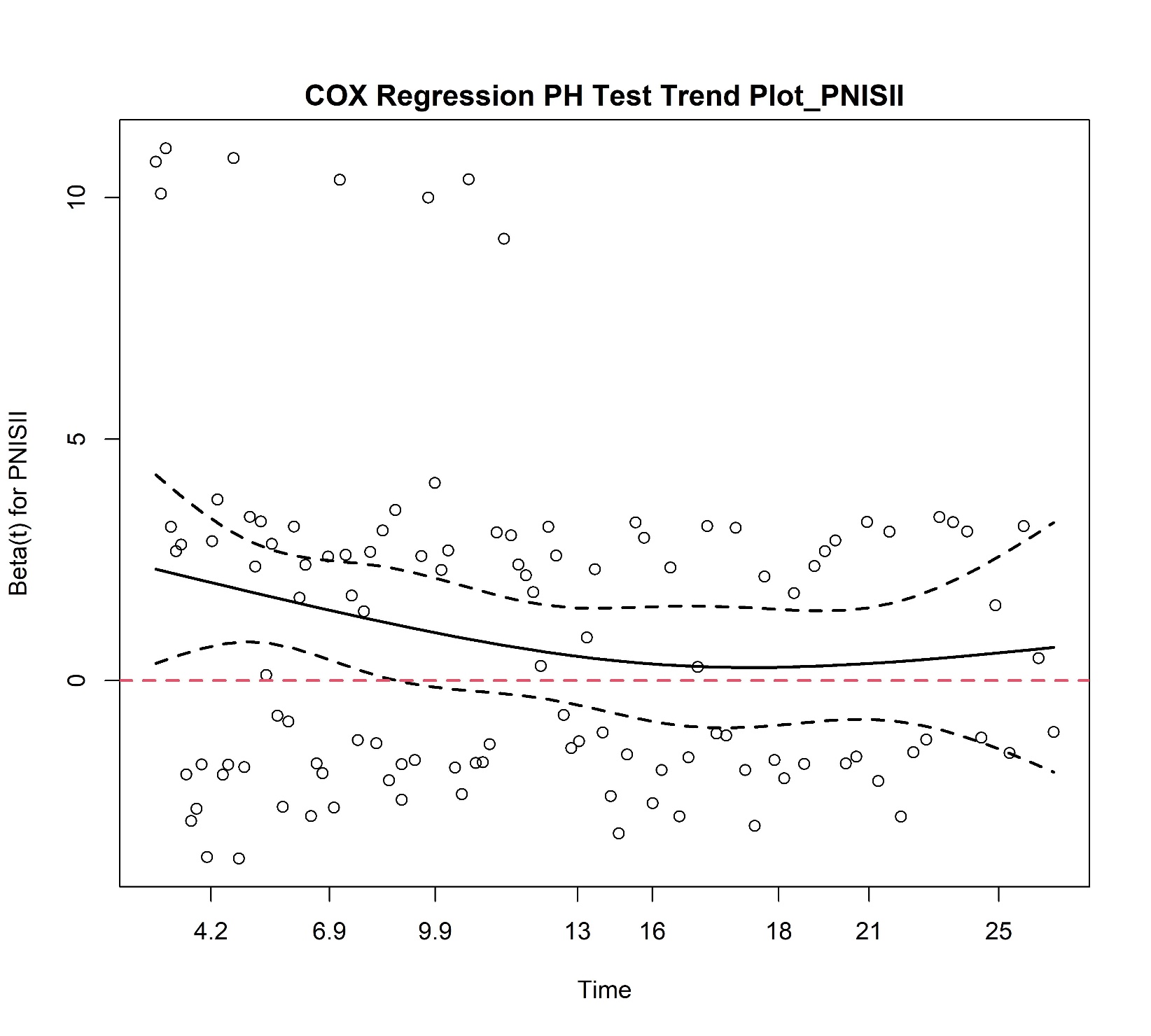
**Figure S6** The trends of the proportional - hazards assumption test based on the COX regression model
